# The SALV-Dataset Registry: An Expertly Curated Digital Clinicopathological Dataset for Salivary Gland Tumor Research and AI-Assisted Diagnostic Tools

**DOI:** 10.1007/s12105-026-01907-1

**Published:** 2026-06-05

**Authors:** Kimberly S. T. Burghout, Jurre A. J. Weijer, Alize C. Evers, Gerben E. Breimer, Jeroen N. van Rossem, Laura A. N. Peferoen, Elisabeth Bloemena, Sjors A. Koppes, Vera van Dis, Robert M. Verdijk, Ilse van Engen-van Grunsven, Thom Doeleman, Mari F. C. M. van den Hout, Bert van der Vegt, Jan Johannes Doff, Hans Marten Hazelbag, Laura A. Smit, Erienne M. V. de Cuba, Marc L. Ooft, Niels J. Rupp, Elisabeth M. P. Steeghs, Mischa de Ridder, Remco de Bree, Marije Slingerland, Vincent T. H. B. M. Smit, Sylvia L. van Egmond, Danielle Cohen

**Affiliations:** 1 Department of Pathology, Leiden University Medical Center, Leiden, The Netherlands; 2 Department of Pathology, University Medical Center Utrecht, Utrecht, The Netherlands; 3 Department of Pathology, Amsterdam UMC, Cancer Center Amsterdam, Amsterdam, The Netherlands; 4 Department of Pathology, Erasmus MC University Medical Center Rotterdam, Rotterdam, The Netherlands; 5 Department of Pathology, Radboud University Medical Center, Nijmegen, The Netherlands; 6 Department of Pathology, GROW-Research Institute for Oncology and Reproduction, Maastricht University Medical Center, Maastricht, The Netherlands; 7 Department of Pathology, Department of Pathology and Medical Biology, University Medical Center Groningen, Groningen, The Netherlands; 8 Department of Pathology, Haaglanden Medical Center, The Hague, The Netherlands; 9 Department of Pathology, the Netherlands Cancer Institute, Antoni Van Leeuwenhoek Hospital, Amsterdam, The Netherlands; 10 Department of Pathology, Location Rijnstate Hospital, Pathology-DNA, Arnhem, The Netherlands; 11 Department of Pathology and Molecular Pathology, University Hospital Zurich, Zurich, Switzerland; 12 Faculty of Medicine, University of Zurich, Zurich, Switzerland; 13 Department of Radiation Oncology, University Medical Center Utrecht, Utrecht, The Netherlands; 14 Department of Head and Neck Surgical Oncology, University Medical Center Utrecht, Utrecht, The Netherlands; 15 Department of Medical Oncology, Leiden University Medical Center, Leiden, The Netherlands; 16 Department of Otorhinolaryngology and Head and Neck Surgery, Leiden University Medical Center, Leiden, The Netherlands; 17 Department of Oral and Maxillofacial Surgery and Oral Pathology, Amsterdam UMC, Amsterdam, The Netherlands; 18 Academic Centre for Dentistry Amsterdam (ACTA), Amsterdam, The Netherlands

**Keywords:** Salivary gland tumors, Interobserver agreement, Diagnostic revision, Artificial intelligence

## Abstract

**Abstract:**

Salivary gland tumors are rare and morphologically diverse, posing both diagnostic and scientific challenges. This study presents the first phase of the SALV-Dataset Registry; a nationwide, expertly curated, and fully digitized clinicopathological resource, designed to support research and develop artificial intelligence (AI) tools assisting in salivary gland tumor pathology diagnostics.

**Methods:**

Salivary gland tumor resections diagnosed at the Leiden University Medical Center (1999–2024) were collected through the Dutch national network and registry for histo- and cytopathology (PALGA). In total, 685 cases were included. Hematoxylin- and eosin-stained slides were digitized and independently reviewed by three teams of head and neck pathologists, in line with the 2023 WHO Classification of Head and Neck Tumours. Discordant and ambiguous cases were resolved in consensus meetings, with access to immunohistochemistry, molecular analysis, and clinical data. Interobserver agreement among the three teams was quantified (Fleiss’ kappa), and agreement between the original and consensus diagnosis was determined (Cohen’s kappa).

**Results:**

Of the 685 tumors, 75% were benign, 24% malignant, and 1% of uncertain malignant potential. The parotid gland was most frequently involved (86%), and the highest rate of malignancy was observed in the sublingual gland (100%). Rare entities were represented, although numbers remained limited. Interobserver agreement was substantial (κ of 0.64; 95% 0.60–0.67). Agreement between the original and consensus diagnosis was excellent (Cohen’s κ of 0.89; 95% CI 0.86–0.92). Forty-three cases (6%) were reclassified following revision, with a change in diagnostic category in 11 cases (2%).

**Conclusion:**

This study demonstrates the feasibility of large-scale, expert digital revision of salivary gland tumors, and establishes a robust foundation for future clinicopathological and AI-based research. With national expansion ongoing, the SALV-Dataset Registry will provide a comprehensive resource for AI training, validation, and clinically oriented modeling in salivary gland tumor diagnostics.

**Supplementary Information:**

The online version of this article (10.1007/s12105-026-01907-1) contains supplementary material, which is available to authorized users.

## Introduction

Salivary gland neoplasms represent a rare and heterogeneous group of tumors comprising of 36 distinct entities described in the 2023 WHO Classification of Head and Neck Tumours. The classification continues to evolve through advances in molecular testing, the identification of novel entities, and the redefinition of existing entities [1–6]. Accurate diagnosis is essential for patient management. Yet, given the evolving classification, overlapping morphological features, and the infrequency with which most pathologists encounter salivary gland tumors, this remains challenging. Reported discordance rates ranging from 8.3 to 29% after expert review, highlighting the diagnostic difficulties [7–9].

Digital pathology and artificial intelligence (AI) may offer practical solutions to improve the accuracy and consistency in the diagnosis of salivary gland tumors. AI models trained on histopathological data have shown to be capable of classifying salivary gland tumors, distinguishing benign and malignant tumors, subtyping of malignant entities, and grading tumors [10–12]. However, existing studies are limited by small, single-center cohorts, and rare subtypes remain underrepresented. The limited availability of large, high-quality digital datasets has constrained both research and AI development in salivary gland tumors.

To address this gap, we initiated the SALV-Dataset Registry, a nationwide initiative to create an expertly curated and fully digitized clinicopathological resource of salivary gland tumors in the Netherlands. This study presents the initial series of 685 resections diagnosed at Leiden University Medical Center (LUMC), each systematically revised and digitized. The reliability of the revision process is demonstrated through analyses of interobserver agreement and diagnostic concordance with the original reports. The present study establishes the foundation for a nationwide registry projected to exceed 5,000 cases, facilitating large-scale clinicopathological research and the development of AI-based diagnostic tools.

## Material and Methods

### Cohort Selection

From 1999 to 2024, all primary and recurrent salivary gland tumor resections analyzed at the LUMC were retrospectively identified through a query of the Dutch national network and registry for histopathology and cytopathology (PALGA), see Supplementary material 1. The query yielded 1,922 cases. Exclusion criteria were applied as follows: (1) non-neoplastic salivary gland resections (e.g. sialoadenitis), (2) biopsy specimens, (3) metastatic neoplasms in the salivary glands, (4) non-epithelial neoplasms (e.g. lymphoma), (5) lymph node resections without salivary gland resection, (6) cytological specimens and/or (7) squamous cell carcinomas (SCC). As a result, the current dataset is limited to surgically resected, primary epithelial salivary gland tumors. SCCs were excluded as these are almost invariably (cutaneous) metastases, and distinguishing primary from metastatic SCC remains extremely challenging [7].

### Digitization of Histological Slides and Quality Control

For each case, hematoxylin- and eosin-stained (H&E) slides containing tumor tissue were collected from the institutional archive. Slides were digitized at 0.25 microns per pixel and scanned using either the Philips Ultra-Fast Scanner (Philips, Eindhoven, the Netherlands) or the PANNORAMIC 480 scanner (3DHISTECH, Budapest, Hungary). The resulting whole slide images (WSIs) were uploaded to Slide Score, an online platform for digital pathology review and case management [8]. During initial quality control, slides with scanning artifacts, inadequate focus, or absent lesional tissue were excluded. When possible, slides affected by artifacts or inadequate focus were rescanned to obtain adequate image quality.

### Diagnostic Revision Process

During an initial pilot phase, 102 cases were jointly reviewed in online meetings. After an evaluation, the workflow was optimized and turned into a stepped approach, allowing an initial independent and digital assessment by three teams, followed by online consensus meetings for discordant and challenging salivary gland tumor cases only, to increase revision efficiency. Sixteen head and neck pathologists from twelve medical centers were organized into the three revision teams (6–8 pathologists per team), balancing institutional backgrounds and level of experience. Cases were revised in batches (typically 100 at a time) and duplicated to create three separate SlideScore environments for each individual team. Within each team, cases were not preassigned but could revised ad hoc by individual pathologists within their team. Importantly, no discussion of joint scoring within the team occurred in this initial revision phase. Following the digital SlideScore revision, each case received three fully independent diagnoses, one from a single pathologist from one of the three teams. This stepped approach increased throughput by allowing pathologists to score cases whenever time permitted, while still retaining independent input from multiple experts through the three-team setup. Based on the H&E slides, all teams assigned each case benign, malignant or uncertain and a WHO classification from a standardized set of diagnoses. The digital platform left room for a differential diagnosis or a request for additional assessment in the expert panel (Supplementary material 2). Reviewing pathologists were blinded to the original pathology report and to the classification assigned by other teams. Optionally a histological grade could also be assigned to a case, however, grading was not performed systematically as the primary aim of this study was to establish a consensus diagnosis.

### Consensus Classification

Cases with complete agreement among the three independent diagnostic teams were assigned a consensus diagnosis. Discordant or uncertain cases (i.e. ≥ 2 diagnoses or diagnostic uncertainty expressed by any team) were reviewed in online consensus meetings involving the participating pathologists. When required for classification, additional information was reviewed, including immunohistochemistry (IHC), molecular analyses, and relevant clinical data. Ancillary investigations were not available to the teams during the initial H&E-only assessment but could be requested during consensus meetings when indicated. IHC was applied in cases with overlapping morphological features, when differential diagnoses could not be resolved on H&E alone. Commonly used markers included p63, CK7, SOX10, S100, and pan-cytokeratin, with specific markers (such as NOR1, DOG1, CTNNB1, and AR) when relevant for the differential diagnosis. Molecular analyses were performed in selected cases where morphology and IHC were inconclusive, most often using the customized Archer FusionPlex Zurich SalvGlandDx panel implemented in our center [9]. When this did not identify a diagnostic alteration, complementary RNA- or DNA-based panels were applied when needed. Final consensus diagnoses were established through structured discussion. In cases where full consensus could not be reached, a differential diagnosis or a descriptive consensus statement was recorded.

### Data Integration and Management

**Fig. 1:**
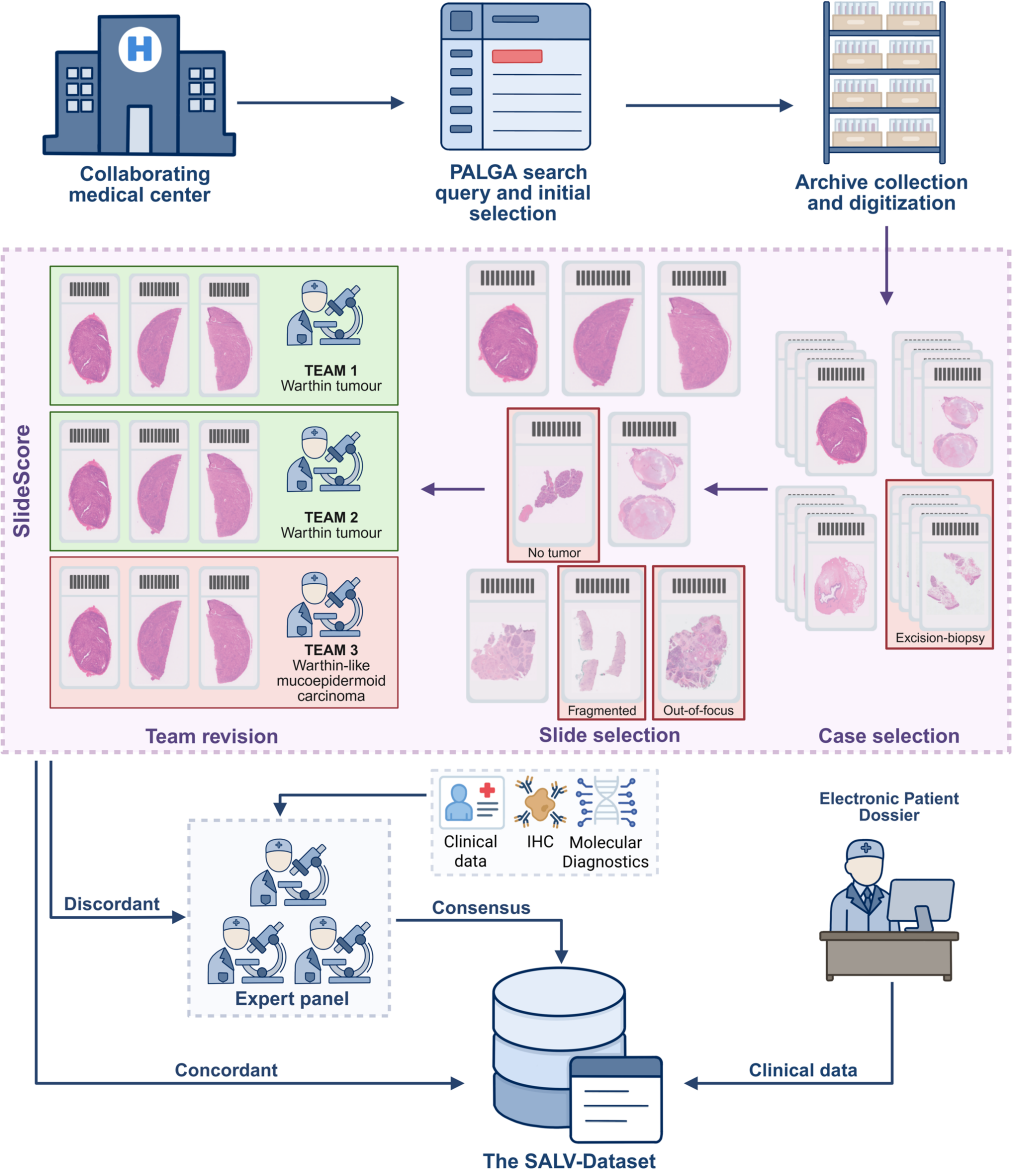
Flowchart of the final SALV-Dataset Registry curation process. Salivary gland tumor resections were identified through a PALGA query and following initial exclusions the corresponding H&E slides were retrieved from institutional archives. Slides were digitized and uploaded to Slide Score, where tumor-containing, high-quality images were selected. Each case was independently reviewed by three expert pathology teams. Concordant diagnoses were accepted, while discordant cases were referred to a central revision panel where consensus was reached using additional clinical information, immunohistochemistry or molecular analyses. Consensus diagnoses, together with slide metadata and clinical parameters, were integrated into the SALV-Dataset Registry. During the initial pilot phase (n = 102) cases were assessed jointly in online meetings before transitioning to independent team-based review system

For each case, clinical metadata were collected, including patient characteristics (age, sex, treatment, follow-up) and tumor characteristics (size, location, histological features). Custom Python scripts using the Slide Score Software Development Kit (SDK) were developed to automate data extraction, perform concordance checks, and integrate final diagnoses into the central dataset, thereby minimizing the risk of manual data entry errors. The curated dataset was managed in Castor within a custom-designed framework that links clinical and pathological data, thereby enhancing usability for current and future analyses. An overview of the full process of data collection and curation is shown in Fig. 1.

### Outcomes and Statistical Analysis

Primary outcomes of the present study were (1) interobserver agreement across three independent revision teams for H&E-based diagnoses, and (2) diagnostic concordance between the original pathology report and final consensus diagnosis, including reclassification rates and changes in diagnostic category (i.e. benign to malignant, malignant to benign). Secondary outcomes included site-specific distributions of salivary gland tumors, frequencies of different tumor types, and identification of the most frequent differential diagnostic considerations. Baseline cohort characteristics were summarized using descriptive statistics. For continuous variables, data were reported as mean ± and standard deviation (SD) when normally distributed, or as median with interquartile range (IQR) when non-normally distributed. Categorical variables, including sex, tumor location, and histological subtype, were expressed as frequencies and percentages. Interobserver agreement across the three independent revision teams was evaluated at the diagnostic level using data exported from three separate Slide Score projects, each corresponding to one team. Analysis of the interobserver agreement was restricted to cases that received three independent diagnoses. Agreement between the independent revision teams was quantified using Fleiss’ κ with corresponding 95% confidence intervals (CI), based on H&E-only diagnoses. Diagnostic concordance between the original and final consensus diagnoses was analyzed by comparing paired results, summarized as the overall discordance rate. Interobserver agreement for pairwise assessment (original versus consensus diagnosis) was further quantified using Cohen’s κ (95% CI) and interpreted according to guidelines by Landis and Koch [15]: < 0.00 = poor; 0.00–0.20 = slight; 0.21–0.40 = fair; 0.41–0.60 = moderate; 0.61–0.80 = substantial; and 0.81–1.00 = almost perfect agreement. All statistical analyses were performed in Python 3.10 using the scikit-learn and statsmodels packages. Descriptive summaries of common differential diagnoses were also provided.

### Ethics Approval

This retrospective study used archived histopathological slides and clinical data from salivary gland tumors to construct a revised digital dataset. In accordance with Dutch law (Medical Research Involving Human Subjects Act, WMO), the study was classified as non-WMO, and the requirement for individual informed consent was waived (reference number nWMO‐D4‐2025‐006). Although informed consent would ordinarily be required, the waiver was granted in view of the retrospective design, the large nationwide cohort, and the implementation of additional ethical safeguards. Patients were informed about the study digitally and through the national salivary gland cancer patient association, with the opportunity to opt out. Because diagnostic re-evaluation could alter previously reported diagnoses, all discordant reclassifications were reviewed by a multidisciplinary panel of clinicians (head and neck surgeons, radiation oncologists, medical oncologists, and specialized head and neck pathologists) to assess potential clinical relevance. Treating physicians and patients were notified when diagnostic changes had implications for current management [16–18]. The study protocol and conduct were reviewed and approved by the (LUMC) non-WMO committee and by institutional ethical, legal, and privacy advisors.

## Results

### Patient and Tumor Characteristics

A total of 685 salivary gland tumor resections from 1999–2024 were included. The mean age at diagnosis was 55 years (SD 15.6), and there was a slight female predominance (n = 377, 55%). Smoking status and alcohol consumption were available for 75% and 73% of patients, respectively. A general overview of patient and tumor characteristics is presented in Table 1.

**Table 1:** Baseline patient and tumor characteristics of the revised salivary gland tumor resections by diagnosis

Consensus diagnosis (n)	Age in years mean ± SD	Sex n; % female	Smoking* n; % active/former	Alcohol n; % active/former	Tumor size in mm median; IQR
Pleomorphic adenoma *n* = *376*	50 ± 16	226 (60%)	121 (34%)	201 (57%)	22 (16–30)
Warthin tumour *n* = *106*	62 ± 9,8	34 (32%)	92 (87%)	68 (65%)	30 (22–40)
Basal cell adenoma *n* = *14*	59 ± 12	6 (43%)	6	7	21 (15–25)
Oncocytoma *n* = *7*	65 ± 8,9	3 (43%)	3	5	17 (11–23)
Lymphadenoma *n* = *3*	61 ± 12,1	3 (100%)	1	1	21 (19–25)
Myoepithelioma *n* = *2*	44 ± 31,8	1 (50%)	–	1	30 (28–31)
Cystadenoma *n* = *2*	53 ± 12,7	0 (0%)	1	1	10 (na)
Striated duct adenoma *n* = *2*	66 ± 1,4	1 (50%)	1	2	14 (13–14)
Canalicular adenoma *n* = *1*	74 ± na	1 (100%)	1	0	10 (na)
Carcinoma ex pleomorphic adenoma** *n* = *31*	61 ± 11,9	14 (45%)	16 (62%)	21 (88%)	25 (20–39)
Adenoid cystic carcinoma *n* = *27*	59 ± 15,4	17 (63%)	8	16 (76%)	24 (14–30)
Mucoepidermoid carcinoma *n* = *25*	54 ± 13,2	16 (64%)	7	9	16 (12–26)
Acinic cell carcinoma *n* = *19*	54 ± 18,4	16 (84%)	6	11 (58%)	25 (15–30)
Salivary duct carcinoma *n* = *16*	70 ± 10,2	4 (25%)	12 (86%)	12 (86%)	25 (19–38)
Polymorphous adenocarcinoma *n* = *11*	61 ± 10,5	6 (55%)	7	7	25 (20–29)
Epithelial-myoepithelial carcinoma *n* = *11*	69 ± 15,1	9 (82%)	6	7	25 (19–26)
Secretory carcinoma *n* = *6*	57 ± 19,5	3 (50%)	3	3	18 (15–19)
Salivary gland carcinoma NOS *n* = *5*	55 ± 7,4	2 (40%)	3	3	27 (25–30)
Hyalinizing clear cell carcinoma *n* = *4*	70 ± 1,7	2 (67%)	1	0	11 (11–24)
Basal cell adenocarcinoma *n* = *3*	72 ± 12,6	1 (33%)	1	2	35 (20–48)
Intraductal carcinoma *n* = *3*	70 ± 15,6	2 (67%)	2	2	13 (11–18)
Myoepithelial carcinoma *n* = *2*	79 ± 5,7	0 (0%)	1	1	41 (36–47)
Mucinous adenocarcinoma *n* = *1*	74 ± na	1 (100%)	0	0	10 (na)
Lymphoepithelial carcinoma *n* = *1*	67 ± na	0 (0%)	0	1	27 (na)
Indeterminate classification *n* = *7*	57 ± 17,2	5 (56%)	3	2	19 (15–22)

*Percentages for smoking and alcohol use are reported only for diagnoses with ≥ 10 cases, otherwise, only absolute numbers are shown.**Subtypes of carcinoma ex pleomorphic adenoma (CXPA) are not shown in this table and are detailed in the Results section

**Fig. 2:**
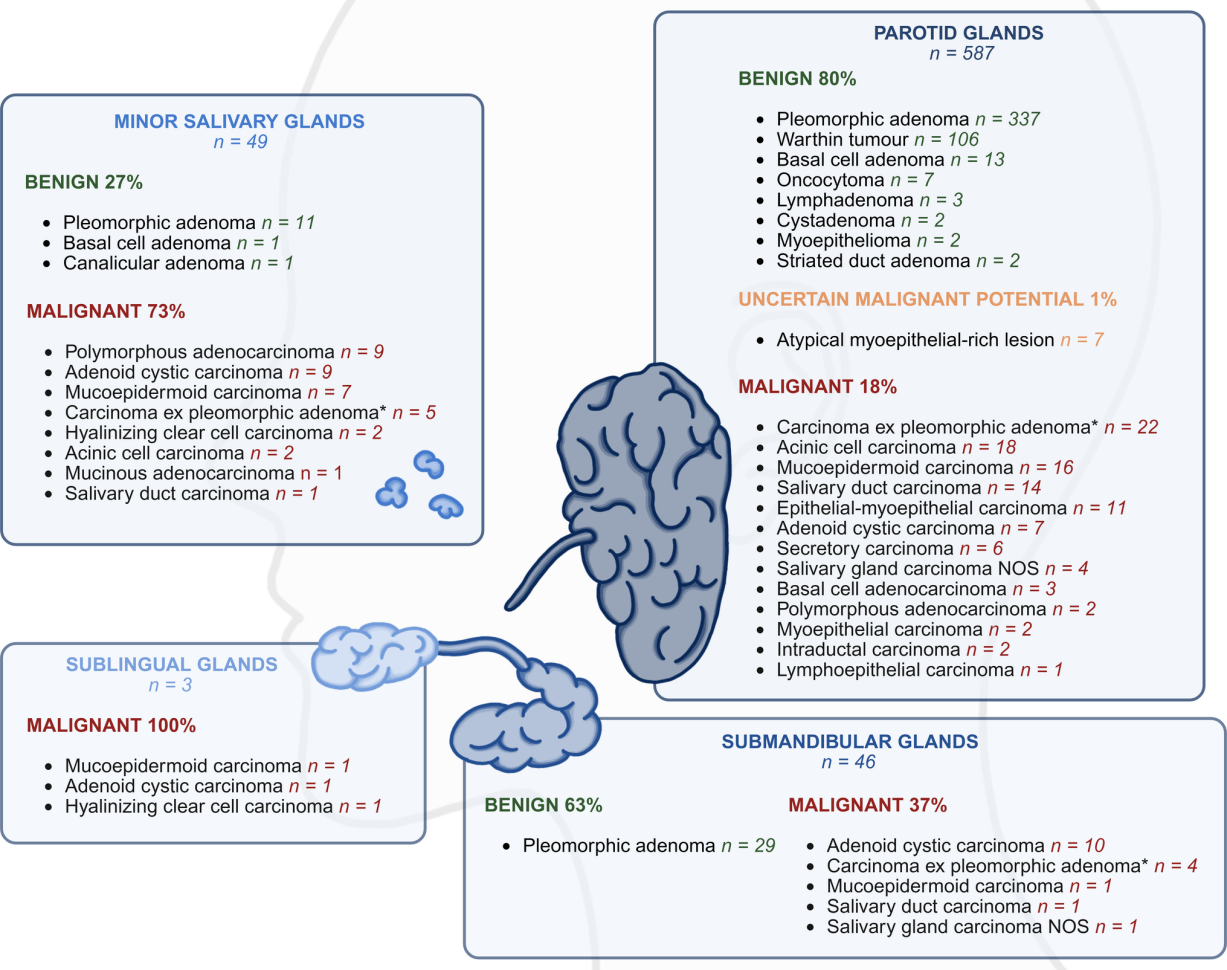
Schematic overview of the distribution of benign and malignant entities across the main anatomical sites. The parotid gland (n = 587) showed mostly benign tumors (80%), most commonly pleomorphic adenomas and Warthin tumors. In the submandibular gland (n = 46), benign tumors accounted for 63% (all pleomorphic adenoma), while adenoid cystic carcinoma was the most frequent malignant tumor. All tumors originating in the sublingual gland (n = 3) were malignant. In the minor salivary glands (n = 49), malignant tumors (73%) predominated, with polymorphous adenocarcinoma and adenoid cystic carcinoma as the most frequent entities

Following revision, 513 tumors (75%) were classified as benign, 165 tumors (24%) as malignant, and 7 tumors (1%) as neoplasm of uncertain malignant potential. The majority of tumors originated from the parotid gland, while the submandibular, minor salivary, and sublingual gland were less frequently involved, as shown in Fig. 2. The most commonly encountered benign entities were pleomorphic adenoma (n = 376, 55%) and Warthin tumor (n = 106, 15%). Among malignancies, carcinoma ex pleomorphic adenoma (n = 31, 5%), adenoid cystic carcinoma (n = 27, 4%), mucoepidermoid carcinoma (n = 25, 4%) and acinic cell carcinoma (n = 19, 3%) were the most often diagnosed tumor types. Carcinomas ex pleomorphic adenoma were further classified by the malignant component, most often salivary duct carcinoma (n = 17) and adenocarcinoma NOS (n = 10), and less frequently myoepithelial carcinoma, epithelial-myoepithelial carcinoma, and one secretory carcinoma. The case containing secretory carcinoma demonstrated a malignant component with predominantly solid and microcystic architecture, and diffuse immunohistochemical staining for S100, SOX10 and CK7. This component was negative for p63 and p40. Centrally, a hyalinized nodule containing residual glandular structures was present, immunohistochemically exhibiting a partial biphasic staining for p63 and p40, highlighting basal cells. This hyalinized nodule was therefore potentially consistent with a pre-existing pleomorphic adenoma. Molecular analysis of the malignant component showed an *ETV6::NTRK3* rearrangement. No *HMGA2* or *PLAG1* rearrangements were detected. Based on the combined morphologic, immunophenotypic and molecular findings, a consensus diagnosis of carcinoma ex pleomorphic adenoma, secretory carcinoma was rendered. However, in the absence of *HMGA2* or *PLAG1* rearrangement, a collision tumor of pleomorphic adenoma and secretory carcinoma could not be entirely excluded.

Molecular analysis was not performed systematically, but applied in diagnostically challenging cases. In total, 121 of 685 tumors (18%) underwent molecular testing, partly during the routine diagnostic workup, and partly upon request during the revision process. Most analyses were conducted using the customized Archer SalvglandDx panel, with complementary RNA- and/or DNA-based assays applied when indicated. The median sizes of benign and malignant tumors were 24 mm (IQR 9–39) and 24 mm (IQR 8–40), respectively.

Seven cases, with histology detailed in Supplementary material 3, were classified as myoepithelial lesions with uncertain malignant potential. This indeterminate classification was based on the combination of multi-lobular growth, close association with surrounding pre-existing salivary gland tissue, cytonuclear atypia (including anisokaryosis and bizarre nuclei), and increased mitotic activity. In all cases, these findings were insufficient to support a diagnosis of carcinoma ex pleomorphic adenoma.

### Histopathological and Clinical Revision

#### Interobserver Agreement

As mentioned, of 685 revised salivary gland tumors, 102 were evaluated prior to implementation of the three-team review structure and were discussed directly in consensus meetings, therefore lacking a team-level diagnoses. Among the remaining 583 cases reviewed by three teams, 10 had incomplete data due to incorrectly submitted forms or technical issues (e.g. out of focus slides at high magnification). Consequently, 573 cases with complete, independent team assessments were available for interobserver analysis. Overall concordance was 68% with a Fleiss κ of 0.64 (95% CI, 0.60–0.67), indicating substantial agreement. The most frequent discordant cases included: (1) pleomorphic adenoma versus pleomorphic adenoma with atypical features vs. carcinoma ex pleomorphic adenoma; (2) pleomorphic adenoma (myoepithelial-predominant) versus myoepithelioma; and (3) adenoid cystic carcinoma versus polymorphous adenocarcinoma versus basal cell adenocarcinoma. An overview of all differential diagnoses is provided in Supplementary material 4.

#### Discordant Consensus Diagnoses

**Fig. 3:**
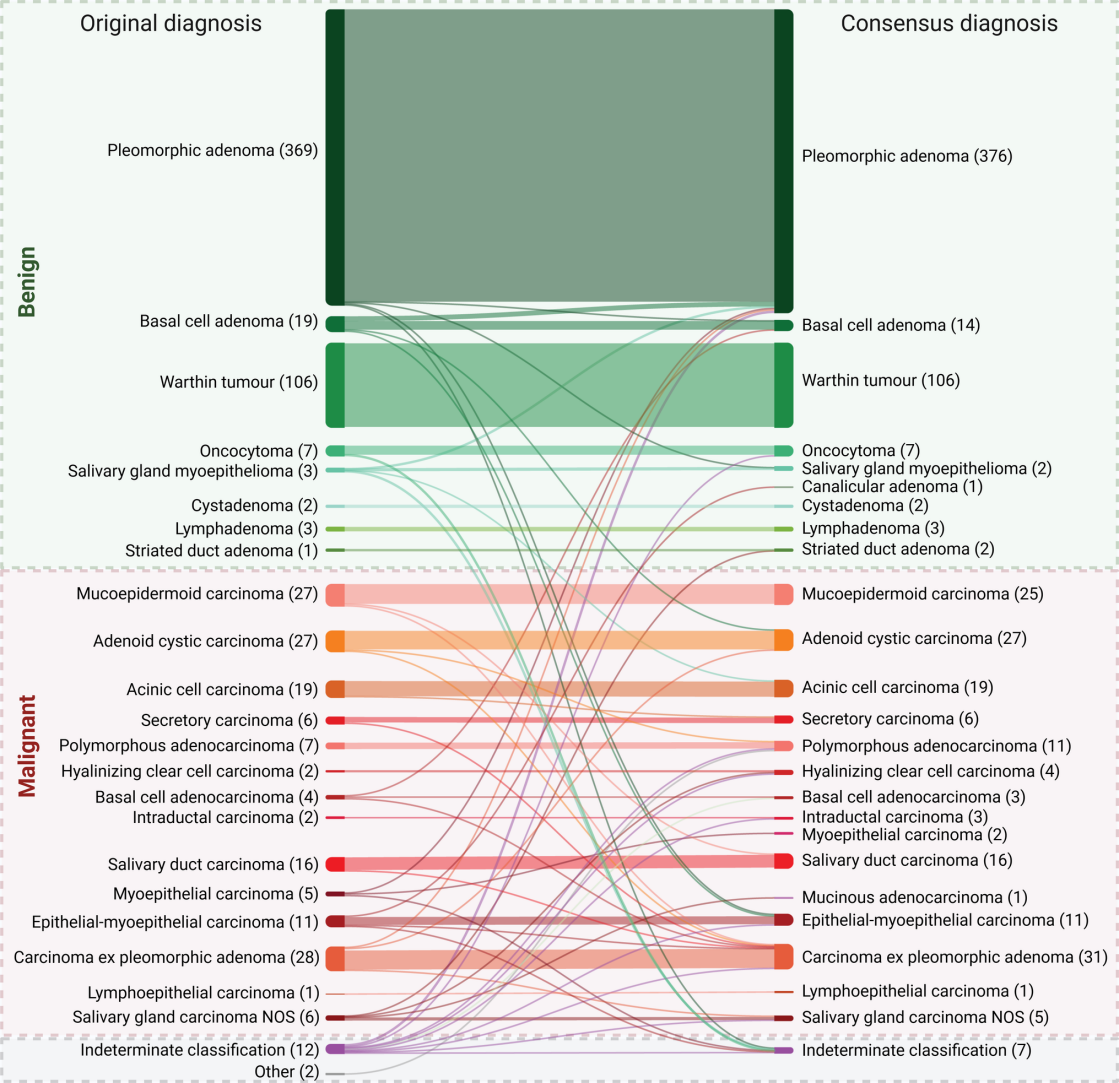
Alluvial plot illustrating the comparison between original pathology diagnoses (left) and consensus diagnoses established through the revision (right) in the SALV-Dataset. Each flow shows the specific original diagnosis and how it was (re-)classified after expert review. The width of each stream corresponds to the number of cases following that diagnostic path

When comparing the original and consensus diagnoses, the overall discordance rate was only 6% (43 of 685), with Cohen’s κ 0.89 (95% CI, 0.86–0.92). The discordance rate was higher among malignancies (11%, 18 of 162) than among benign tumors (3%, 17 of 514). Some diagnostic discrepancies reflected updates in WHO classification over the years, as certain entities were not yet recognized at the time of the original diagnosis, and renamed entities were not considered discordant. A schematic overview of the diagnostic changes is shown in the alluvial plot in Fig. 3.

**Fig. 4:**
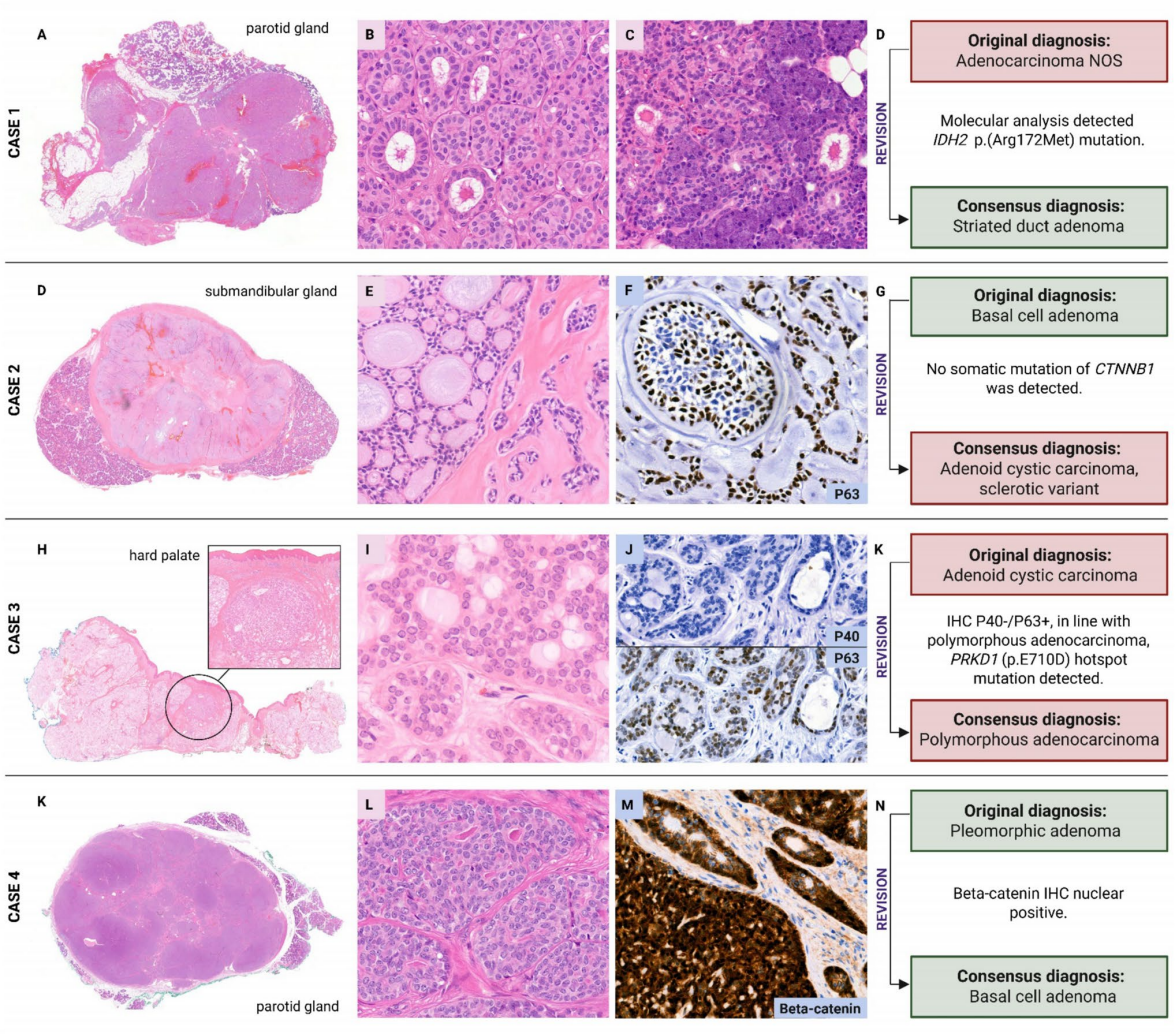
Examples of cases with a diagnostic change following expert revision. Case 1, originally diagnosed as adenocarcinoma NOS, was revised to striated duct adenoma after the detection of an IDH2 p.(Arg172Met) mutation. Case 2, originally diagnosed as basal cell adenoma, was revised to adenoid cystic carcinoma (sclerotic variant) based on morphology, specifically the presence of cytonuclear atypia. No molecular alterations were detected, specifically no CTNNB1 mutation, MYB/MYBL1 gene rearrangement, or MYB RNA overexpression in the Archer analysis. Case 3, originally diagnosed as adenoid cystic carcinoma, was revised to polymorphous adenocarcinoma based on morphology, pattern of p40/p63 immunohistochemistry, and detection of the characteristic PRKD1 hotspot mutation (p.710D). Case 4, originally diagnosed as pleomorphic adenoma, was revised to basal cell adenoma following nuclear β-catenin immunoreactivity

A change in recorded tumor category was observed in only 11 cases (1.6%), including five cases reclassified from benign to malignant, and six cases from malignant to benign. Illustrative cases are presented in Fig. 4. Reclassifications were most often driven by reinterpretation of morphology, supported where needed by IHC and/or molecular testing. The rationale for diagnostic changes in all 43 discordant cases is presented in Supplementary material 5.

#### Clinical Consequence

A multidisciplinary panel reviewed all 43 discordant cases. In 9 cases, the reclassification was considered clinically relevant for ongoing clinical management and required adjustments to the established follow-up, which were communicated to the treating physicians and patients. Although 11 tumors were changing diagnostic categories, not all of these changes had practical clinical implications: in some instances, patients were already deceased, had long-term recurrence-free follow-up, or the management and surveillance strategies were similar for both diagnostic categories.

## Discussion

**Fig. 5:**
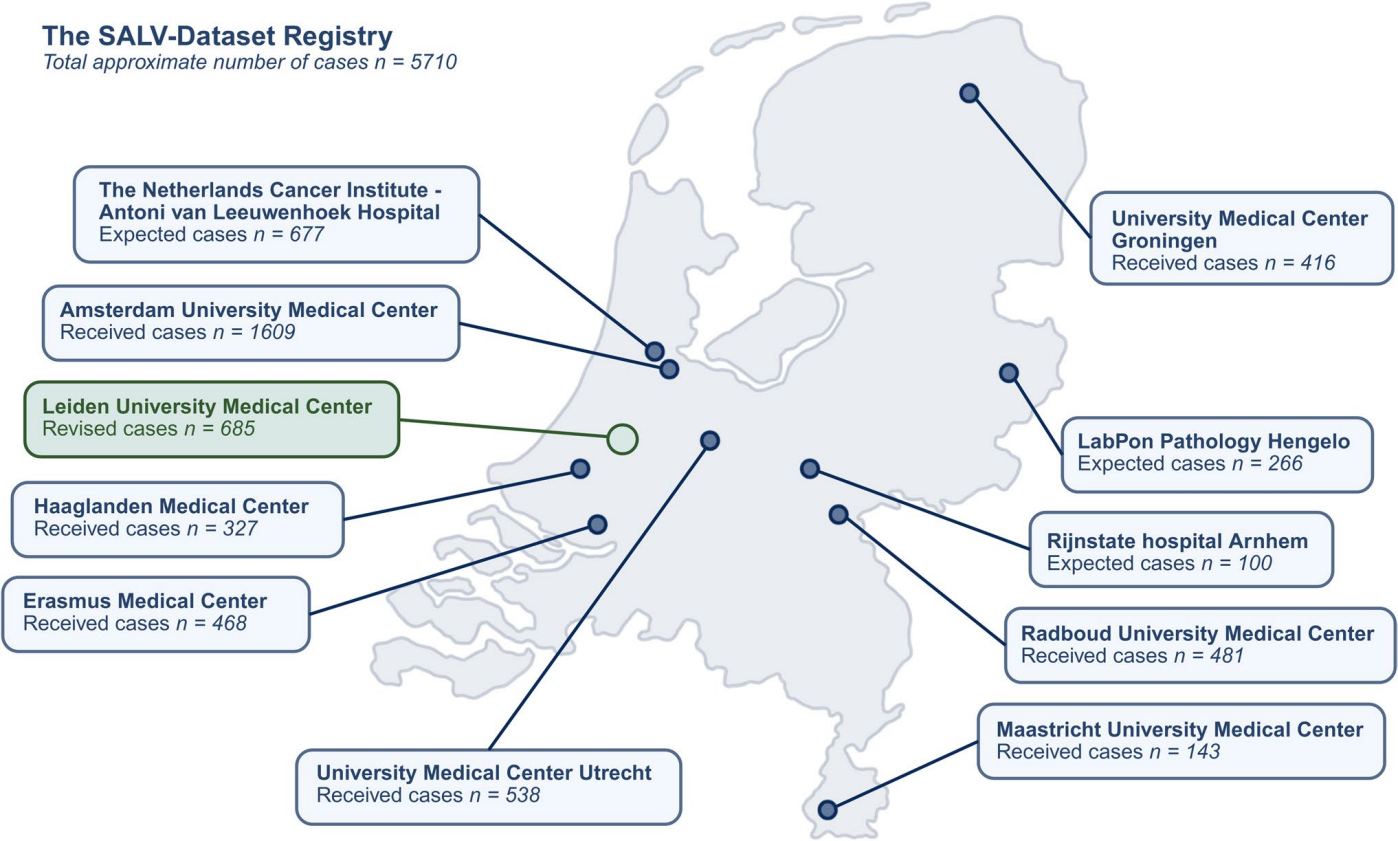
Medical centers currently contributing salivary gland tumors to the SALV-Dataset Registry. Numbers represent the cases received from each collaborating center. The digitalization and revision of the LUMC cases are described in this study; this process is ongoing for the other centers

This study presents the first steps toward constructing a large, fully revised digital dataset for salivary gland tumors, the “SALV-Dataset Registry”. We aim to create a robust foundation that represents all salivary gland tumor entities, revised by a large team of head and neck pathologists. This registry is currently expected to contain 5,000 salivary gland tumor resections derived from eleven medical centers across the Netherlands (Fig. 5). To our knowledge, it represents the first multicenter initiative consisting of a fully digitized series of salivary gland tumor resections, established to support clinicopathological research and facilitate the development of AI-based diagnostic tools. Notably, in this study some of the observed diagnostic discrepancies reflect reclassifications of salivary gland tumors over time [10]. These diagnostic changes are not unexpected given the continuous evolution of WHO classification, and the growing use of immunohistochemistry and molecular testing. Systematic re-evaluation incorporating these contemporary insights is therefore essential to establish a reliable diagnostic ground truth for future AI development and further research.

Within this initial series of 685 digitized and revised salivary gland tumor resections, the stepwise revision process proved feasible, and a consensus diagnosis was obtained in 99% of cases. The distribution of tumor types in the cohort is consistent with prior demographic studies, with an overall malignancy rate of 24% [11–14]. Interestingly, myoepithelioma accounted for only 0.3% of cases in this cohort, compared to approximately 2% in earlier reports, likely reflecting a diagnostic preference to classify cellular myoepithelial lesions as pleomorphic adenoma with predominant myoepithelial component when glandular structures are present [13, 15]. This is consistent with recent molecular evidence placing myoepithelioma and pleomorphic adenoma within a single epigenetic spectrum [16]. Carcinoma ex pleomorphic adenoma was more common in this series (19%) than in previous cohorts (range of 9–11%), potentially reflecting the rigorous revision process that recognized a morphological background of pleomorphic adenoma. In accordance with previous reports, the rate of malignancy was highest in the sublingual glands (100%) and minor salivary glands (72%), and this contextual risk of malignancy is an important diagnostic feature, also valuable in training AI-based diagnostic tools [13, 17]. Clinical metadata, including smoking status, were also collected as smoking is a known risk factor for Warthin tumor, but evidence for an association with malignant salivary gland cancers remains limited [18, 19]. In this cohort, a high prevalence of smoking was observed among patients with salivary duct carcinoma (86%), a finding warranting further investigation in larger cohorts.

Several diagnostic challenges were encountered in the revision process, particularly among tumors with oncocytic, basaloid, or clear-cell morphology, features known to overlap between entities and introduce interobserver variability [20–23]. In many of these cases, consensus could be reached only after integrating clinical, immunohistochemical, and molecular findings, underlining the importance of a structured, stepwise diagnostic approach (highlighted in Supplementary Material 5). Interobserver agreement between the three independent review teams was substantial (Fleiss’ κ = 0.64, 95% CI 0.60–0.67, overall concordance 68.1%). Fuoco et al. (2023) reported a similar moderate agreement (Fleiss’ κ 0.575), albeit in a smaller, morphologically more challenging case series [24]. Following revision, discordance between the original and revised diagnosis was found in 6% of cases (Cohen’s κ = 0.89 (95% CI 0.86–0.92), which is at the lower end of previously reported rates ranging from 8.3 to 29% [25–28]. Clinically, nine discordant diagnoses were considered relevant for current patient management (e.g. follow-up alteration), and following multidisciplinary panel discussion, these conclusions were communicated to the treating physicians and patients. Although uncommon in retrospective studies, communicating these clinically relevant findings was considered ethically justified following consultation with patient associations and ethical advisors.

**Fig. 6:**
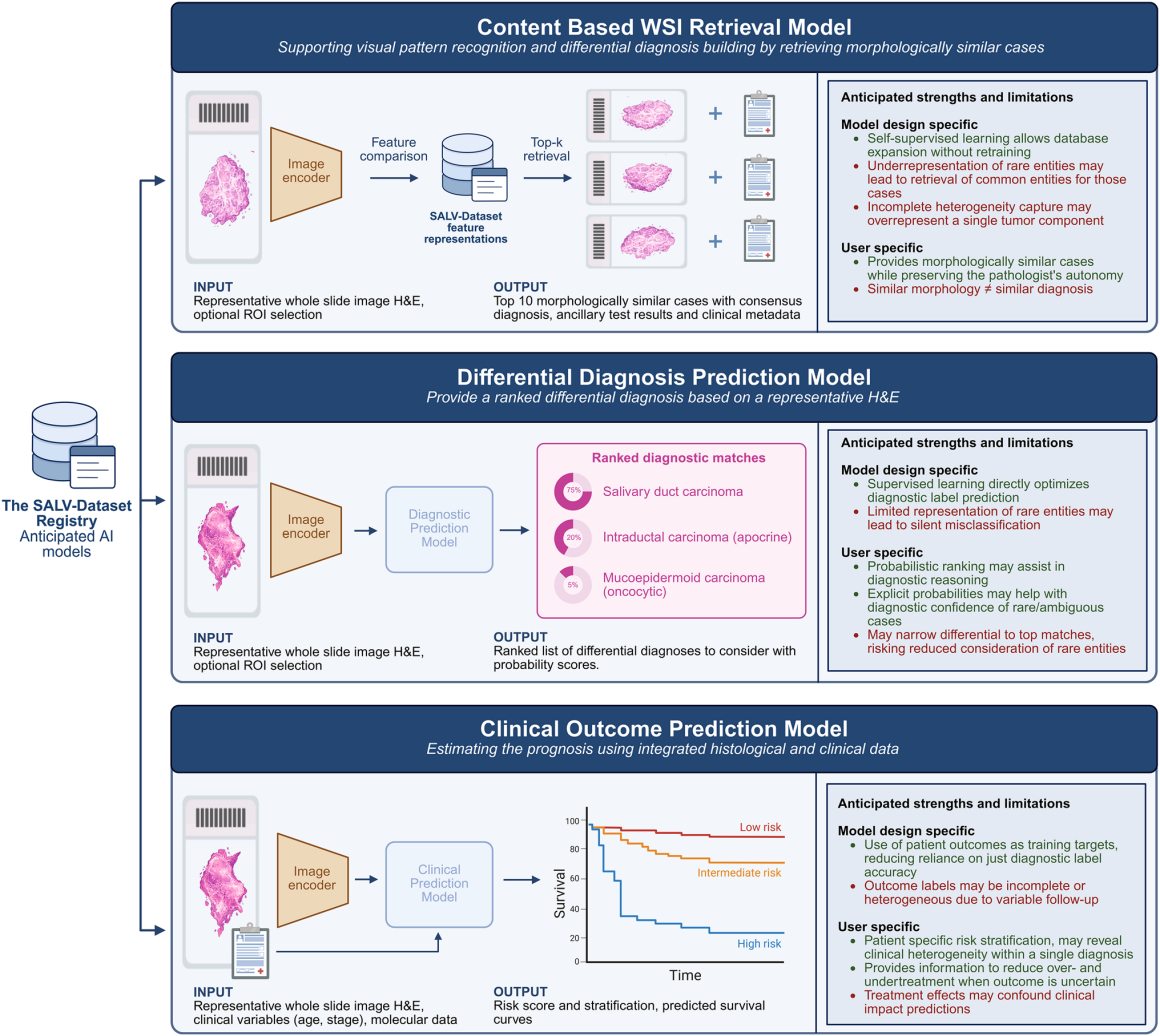
Anticipated AI applications based on the SALV-Dataset Registry. The SALV-Dataset Registry (left) is the foundation of three AI-based decision support approaches in salivary gland tumor pathology. **Top:** a content-based WSI retrieval model retrieves morphologically similar reference cases with associated metadata to support pathologist interpretation. **Middle:** a supervised differential diagnosis prediction model that produces a ranked probabilistic shortlist of matching diagnoses **Bottom:** clinical outcome prediction models integrate histology with clinical (and potentially molecular) data to estimate risk stratification and prognosis

This dataset was created with the explicit goal of enabling AI development, inspired by the substantial momentum AI has gained in pathology, while its use for salivary gland tumors has remained comparatively underexplored. To date, only a limited number of studies have applied machine learning and/or deep learning as the rarity of many salivary gland tumor entities make assembling sufficiently large training cohorts challenging [29–35]. From published works, some learn directly from entire whole-slide images, but frame the task as a pairwise distinction between only two tumor types [31, 32]. Others use region-of-interest-based workflows with manual region selection from which morphometric or color features are obtained using cell segmentation to train interpretable models for benign/malignant distinction, malignant subtyping, and grading [36]. Each of these paths carries a trade-off, either a narrow label scope or reliance on manually curated regions. In parallel, others apply full-slide deep learning, using either transformer models that classify patches or convolutional neural networks with multiple-instance learning to obtain slide-level classifications [29, 30]. Using the SALV-Dataset Registry, we plan multiple deep-learning approaches that extend beyond the strategies explored in prior work. A high-level overview of these approaches, together with their anticipated strengths and limitations is highlighted in Fig. 6.

Despite the recent advances of diagnostic AI-tools, progress remains limited by small, single-center cohorts and the absence of systematically revised, consensus-labeled data [37]. Because cohorts are small and these tumors are rare, uncommon subtypes are underrepresented or excluded, which reduces clinical applicability. Clinical metadata are seldom incorporated, leaving prognostic and treatment-oriented modeling largely unexplored. The SALV-Dataset Registry addressed these gaps, as the dataset includes diagnostically challenging cases and curated clinical metadata. The ongoing case collection and revision are expected to ensure representation of all recognized tumor entities, including rare subtypes. While algorithm development is a primary goal of the SALV-Dataset Registry, the resulting nationwide collection of fully revised, clinicopathologically annotated salivary gland tumors also constitutes a unique research resource, with applications extending beyond AI development. To facilitate access to the registry and support broad national and international collaboration and salivary gland tumor research, a structured legal framework was developed. A formal joint data registry agreement enables (future) participating institutions to access the dataset for collaborative research and facilitate data sharing for answering their own specific research questions. This balances scientific accessibility and dataset expansion while safeguarding patient privacy and local regulations.

This study has several limitations. First, although each case was independently reviewed by three head and neck pathologists, a residual risk of false concordance based on H&E only cannot be fully excluded. The use of multiple independent teams of pathologists with a balanced distribution of experience was considered a reasonable compromise to minimize this risk, while maintaining a practical study design. Second, consensus meetings were essential for discordant cases, though the online format initially limited a detailed morphological assessment. To address this, a structured presentation was used, with annotated H&E and IHC images, clinical and molecular details, and individual access to full-digitized H&E slides to facilitate real-time review. Discussions followed this consistent format for each case, with contributions from all members encouraged regardless of experience, and the relevant literature actively shared. Third, the cohort spans more than two decades during which diagnostic criteria, staining protocols, and scanning technologies have evolved. While this introduces heterogeneity, it also mirrors real-world diagnostic practice and exposes AI models to the unavoidable variability and artifacts. Finally, at this stage, the dataset is derived from a single institution, reflected in an underrepresentation of some salivary gland tumor entities. Expansion into a nationwide, multicenter registry is, as mentioned, ongoing and will address this limitation by increasing case numbers.

In conclusion, the SALV-Dataset Registry demonstrates the feasibility of building a large, high-quality digital resource for salivary gland tumors. With ongoing (inter)national expansion, it will provide a critical platform for robust AI development and validation, ultimately supporting more accurate diagnostics and collaborative research in these often rare and heterogeneous entities. Importantly, the success of AI in salivary gland tumor diagnostics depends entirely on the quality and quantity of the revised and digitized cases and will require large collaborative efforts. 

## Supplementary Information

Below is the link to the electronic supplementary material.

Supplementary file 2 (TIFF 254 kb)

Supplementary file 1 (DOCX 16 kb)

Supplementary file 3 (DOCX 14533 kb)

Supplementary file 4 (DOCX 555 kb)

Supplementary file 5 (DOCX 24 kb)

## Data Availability

The generated data are not publicly available to protect patient privacy, but may, in part, be made available upon reasonable request of the corresponding author.

## Abbreviations

H&E: Hematoxylin and eosin
IHC: Immunohistochemistry
FNA: Fine-needle aspiration
AI: Artificial intelligence
