# Supplementary figures and images for: The SALV-Dataset Registry: An Expertly Curated Digital Clinicopathological Dataset for Salivary Gland Tumor Research and AI-Assisted Diagnostic Tools
Source: Head Neck Pathol. 2026 Jun 5;20(1):62. doi: 10.1007/s12105-026-01907-1 (PMC13241568; doi:10.1007/s12105-026-01907-1)

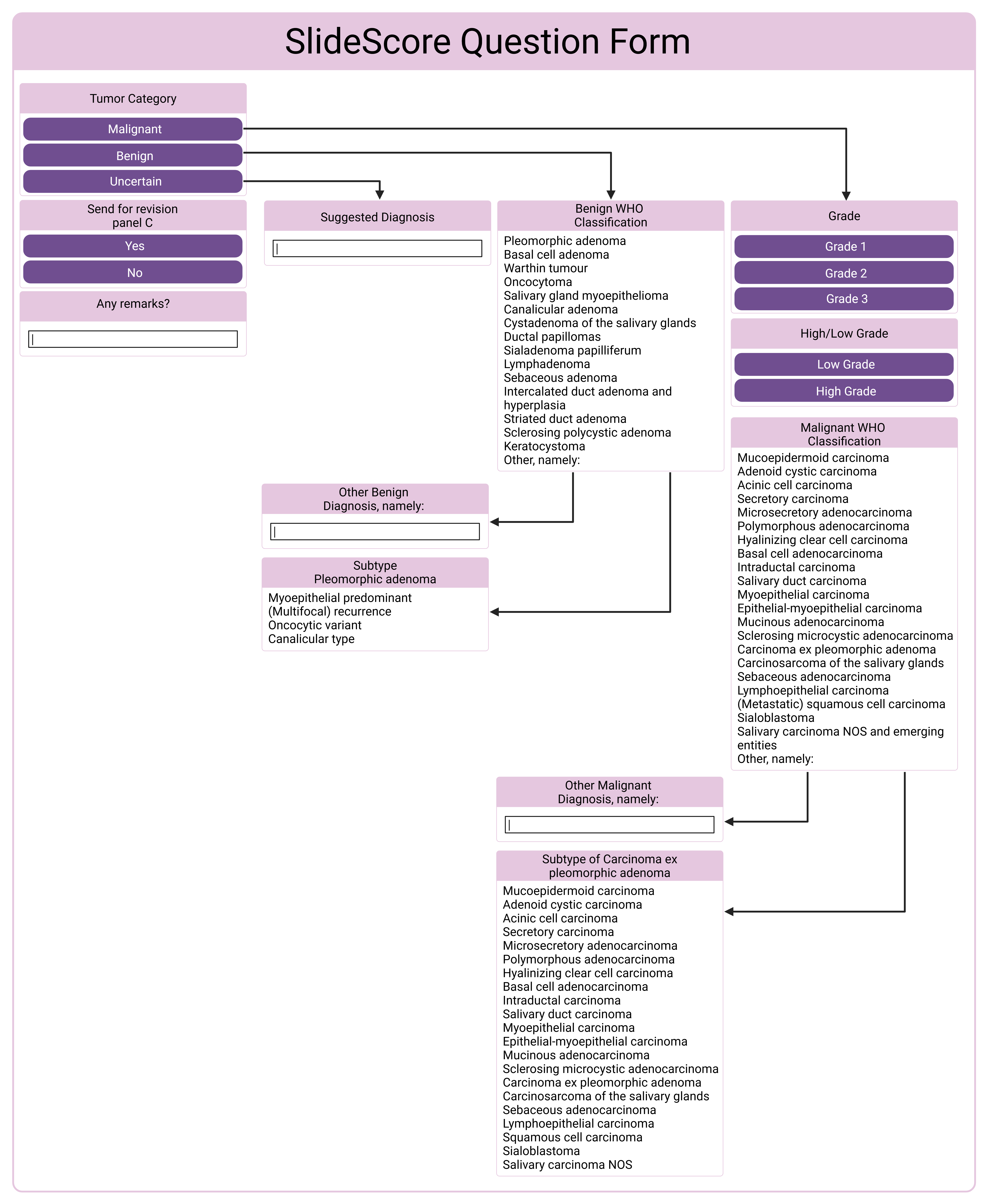

Supplement: Supplementary file 1 — Supplementary file 2 (TIFF 254 kb) [file 12105_2026_1907_MOESM1_ESM.tiff]
