# Supplementary material for: The SALV-Dataset Registry: An Expertly Curated Digital Clinicopathological Dataset for Salivary Gland Tumor Research and AI-Assisted Diagnostic Tools
Source: Head Neck Pathol. 2026 Jun 5;20(1):62. doi: 10.1007/s12105-026-01907-1 (PMC13241568; doi:10.1007/s12105-026-01907-1)
Supplement: Supplementary file 2 — Supplementary file 1 (DOCX 16 kb) [file 12105_2026_1907_MOESM2_ESM.docx]

**Supplementary material 1**

Query of the Dutch national network and registry for histopathology and cytopathology (PALGA) contained the following items:

- All WHO-recognized benign entities including pleomorphic adenoma, Warthin tumor, salivary gland myoepithelioma, basal cell adenoma, oncocytoma, lymphadenoma, cystadenoma, sialadenoma papilliferum, ductal papilloma, sebaceous adenoma, canalicular adenoma, striated duct adenoma, intercalated duct adenoma, sclerosing polycystic adenoma and keratocystoma.
- All WHO-recognized malignant entities including mucoepidermoid carcinoma, adenoid cystic carcinoma, acinic cell carcinoma, polymorphous adenocarcinoma, hyalinizing clear cell carcinoma, basal cell adenocarcinoma, intraductal carcinoma, adenocarcinoma NOS, salivary duct carcinoma, myoepithelial carcinoma, epithelial-myoepithelial carcinoma, carcinoma ex pleomorphic adenoma, secretory carcinoma, lymphoepithelial carcinoma, mucinous adenocarcinoma, sclerosing microcystic adenocarcinoma, carcinosarcoma, sebaceous adenocarcinoma, squamous cell carcinoma and microsecretory adenocarcinoma.
- Salivary gland tumor resection with uncertain diagnosis or dated terminology (e.g. clear cell carcinoma, oncocytic carcinoma)*.

**The search strategy included historical and broader diagnostic terms to account for changes in diagnostic coding over time within the PALGA registry, as this cohort spans 25 years. These terms were used solely to optimize case retrieval.*
