# Supplementary material for: The SALV-Dataset Registry: An Expertly Curated Digital Clinicopathological Dataset for Salivary Gland Tumor Research and AI-Assisted Diagnostic Tools
Source: Head Neck Pathol. 2026 Jun 5;20(1):62. doi: 10.1007/s12105-026-01907-1 (PMC13241568; doi:10.1007/s12105-026-01907-1)
Supplement: Supplementary file 3 — (DOCX 14533 kb) [file 12105_2026_1907_MOESM3_ESM.docx]

**Supplementary material 3**

Histological images of lesions with uncertain malignant potential.

*Myoepithelial lesion with atypical features (case T11-00273)*

**
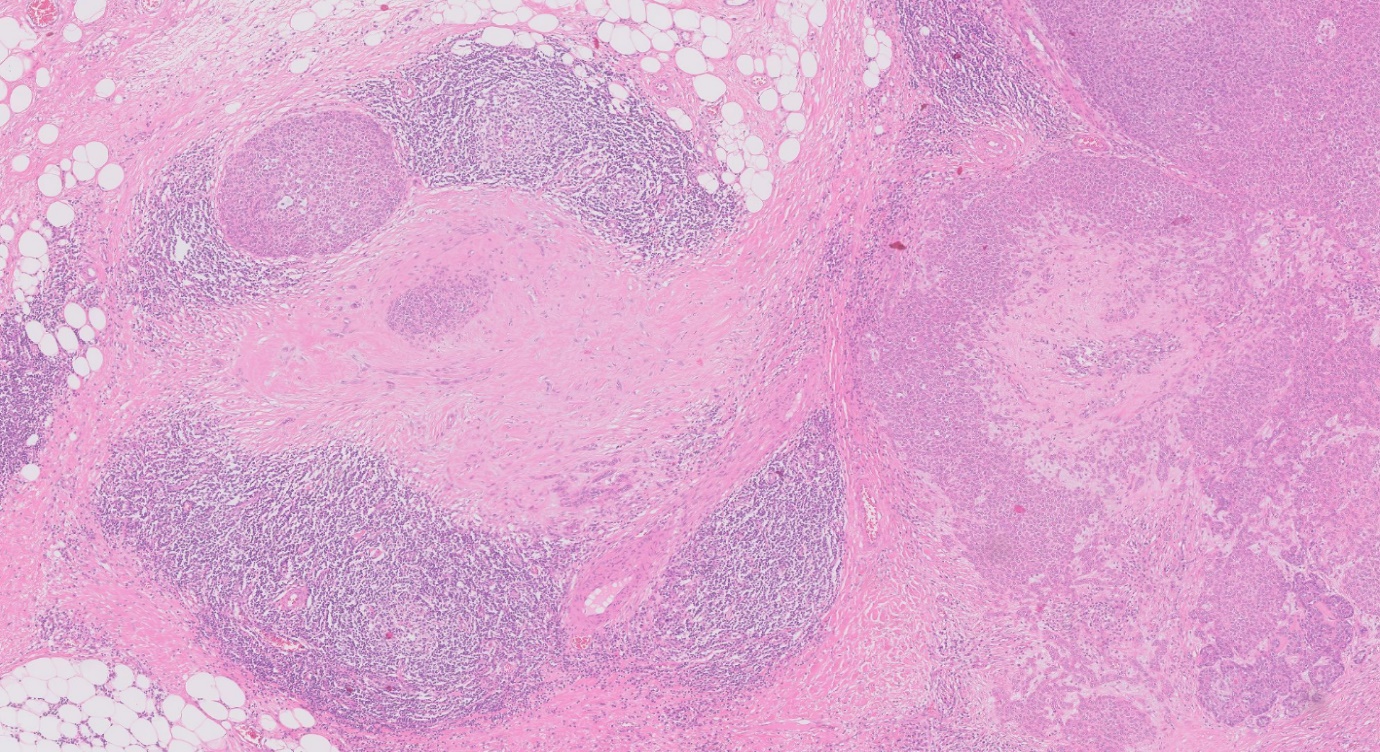

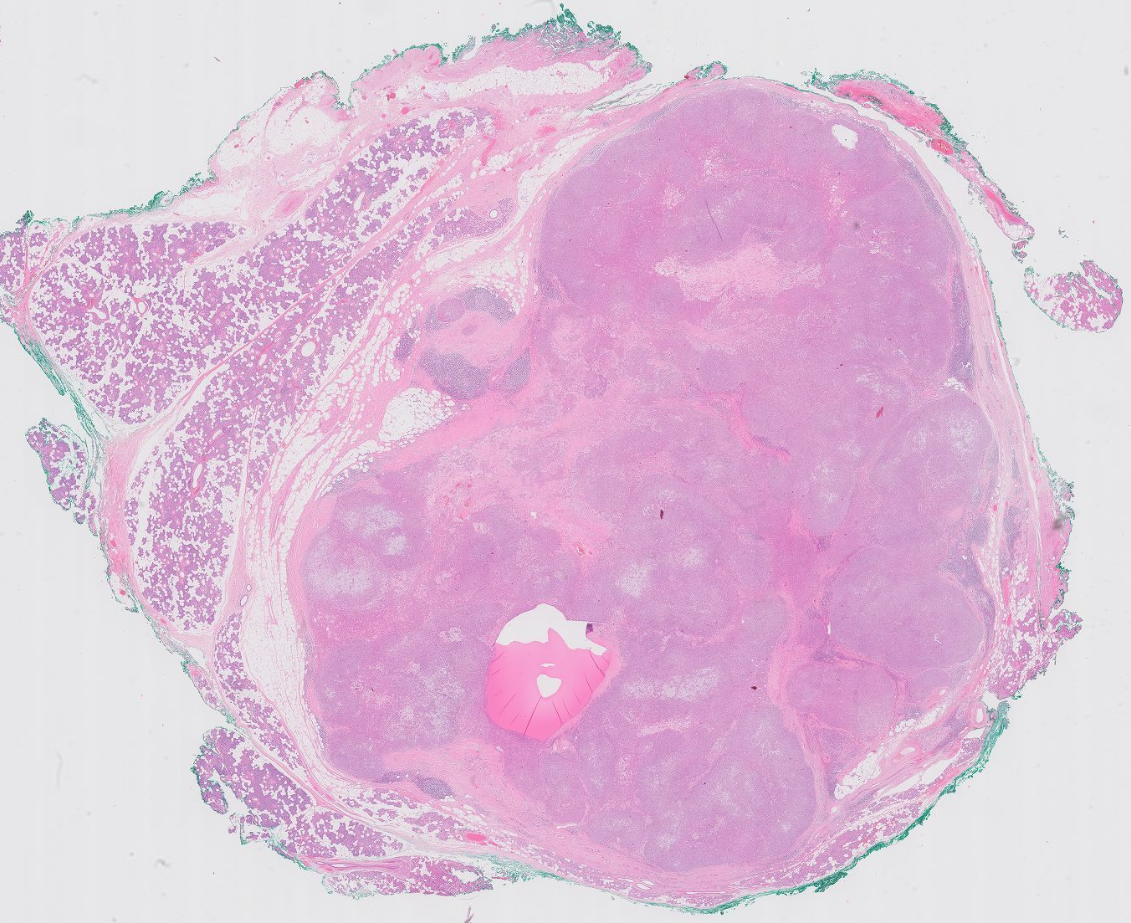
** **
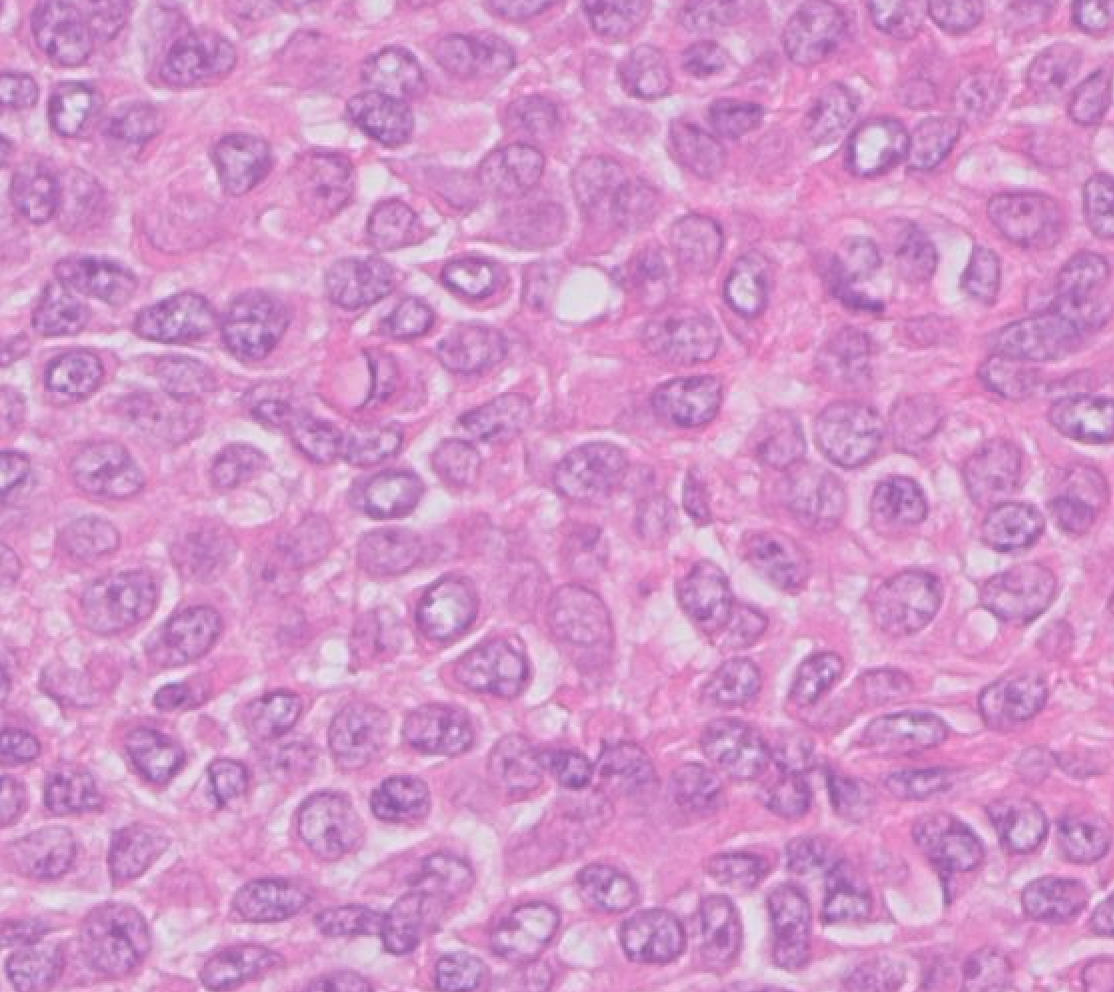
**

*Myoepithelial lesion with atypical features (case T11-00427)*

**
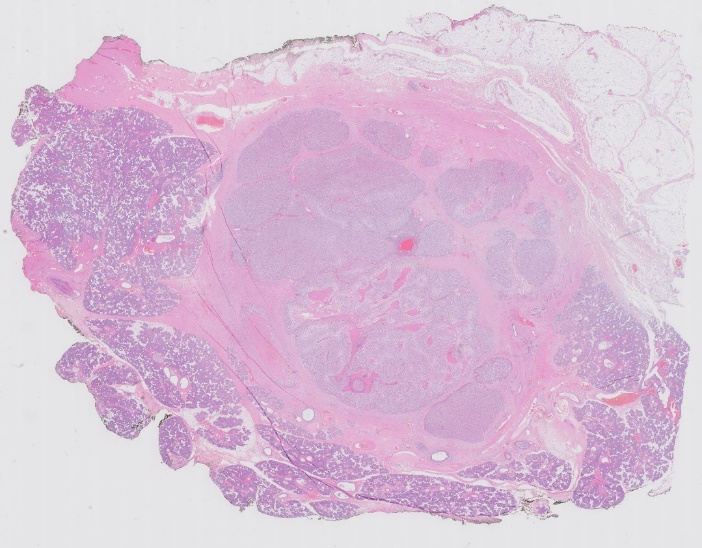
** **
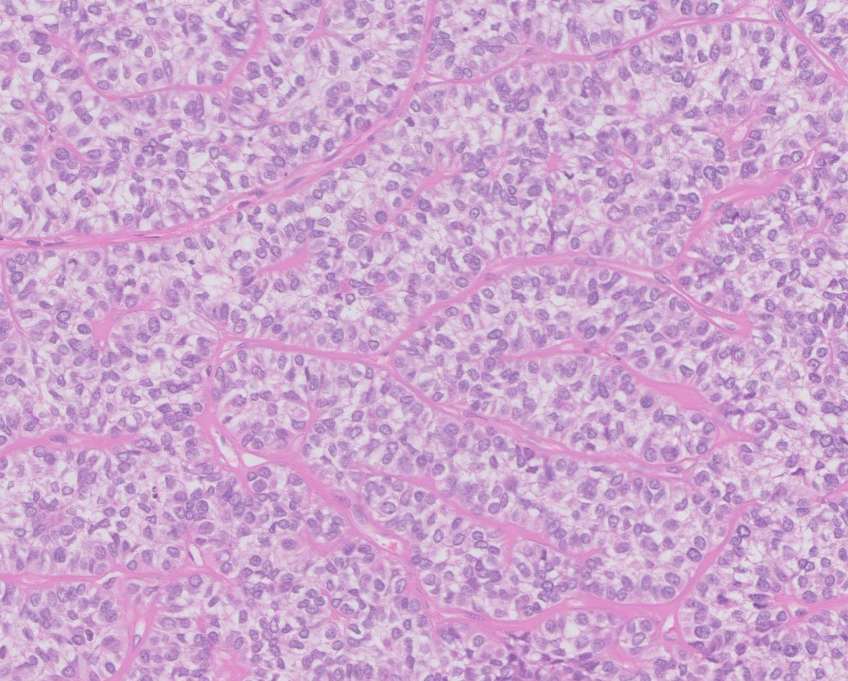
**

*Myoepithelial lesion with atypical features (case T11-00875)*

**
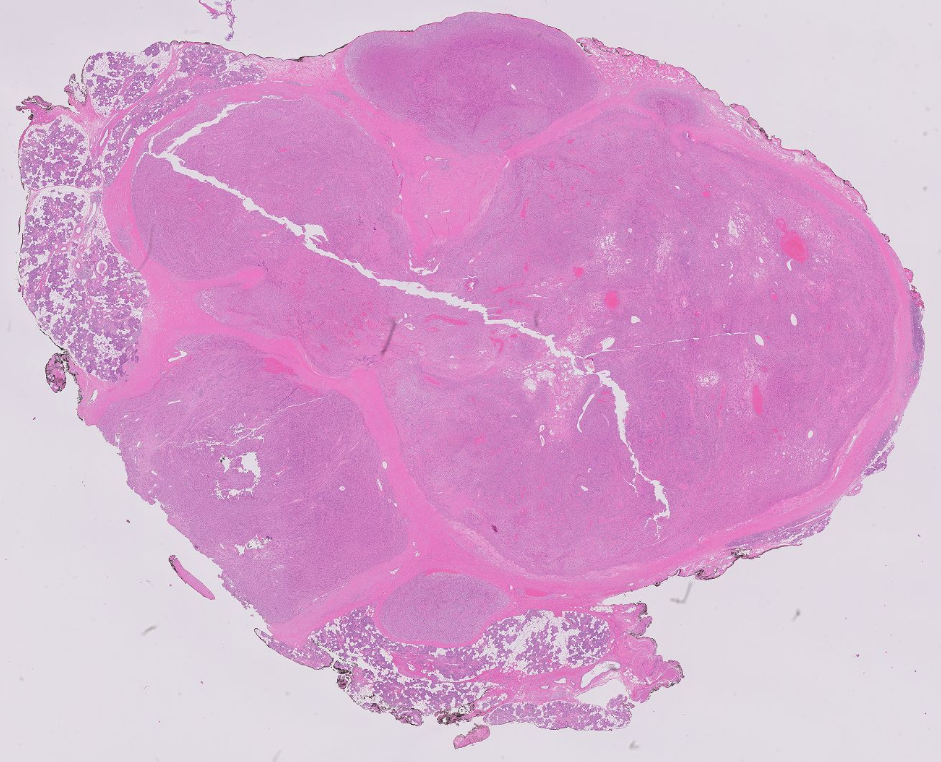
** **
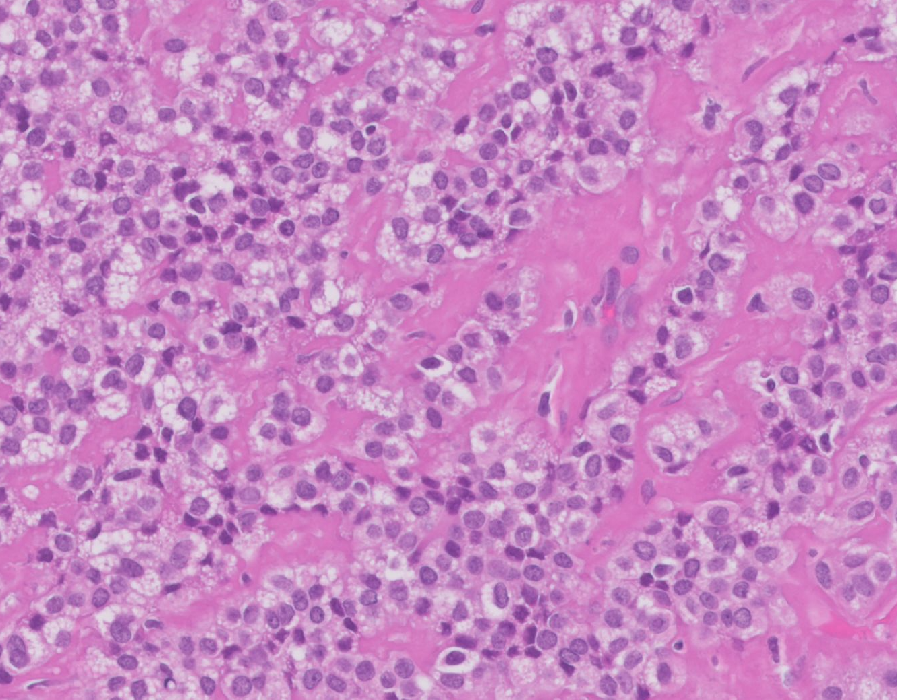
**

*Myoepithelial lesion with atypical features (case T11-00903)*

**
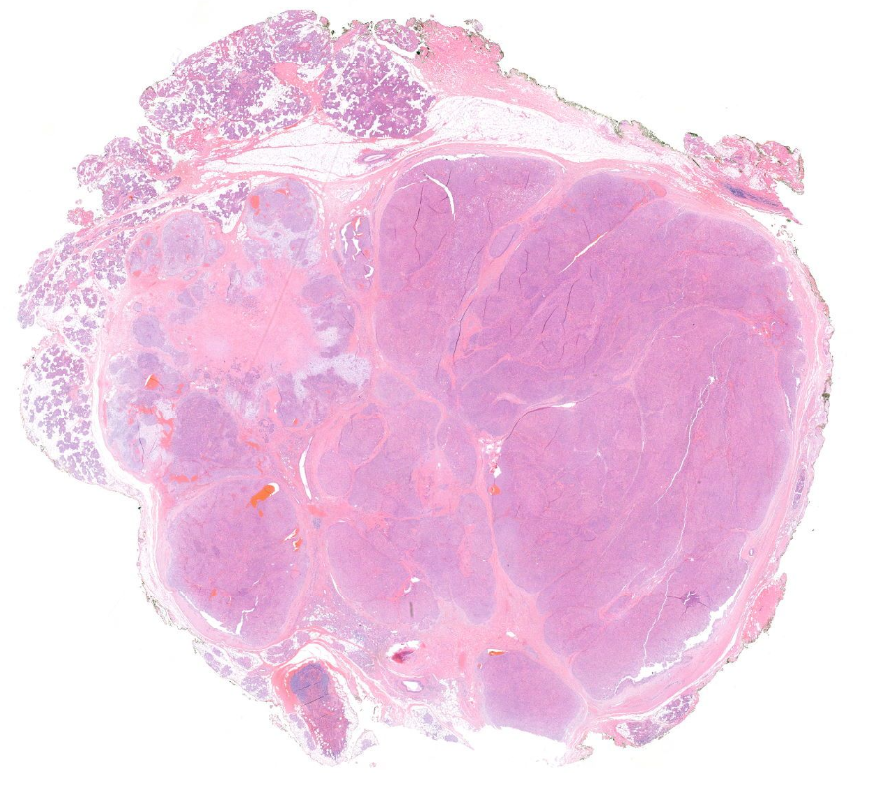
** **
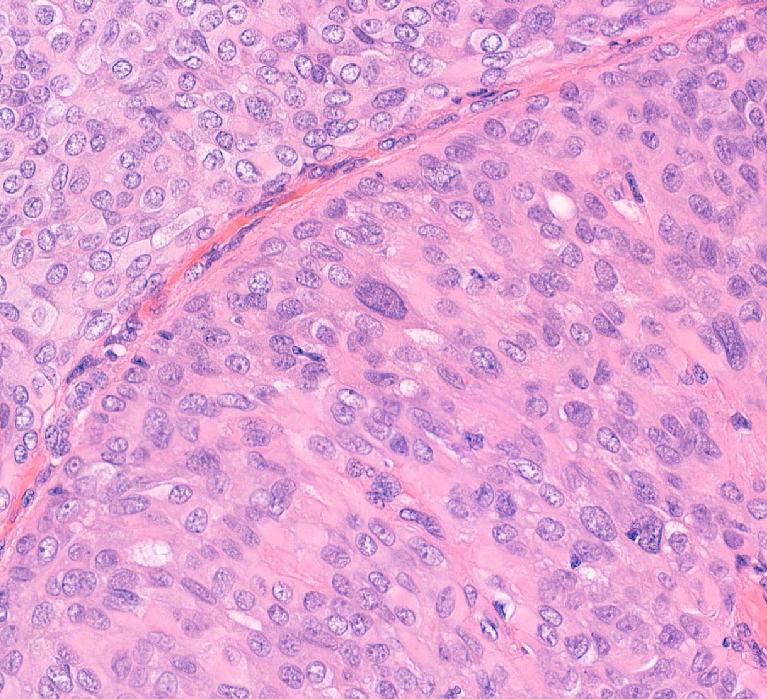
**

*Myoepithelial lesion with atypical features (case T11-00972)*

**
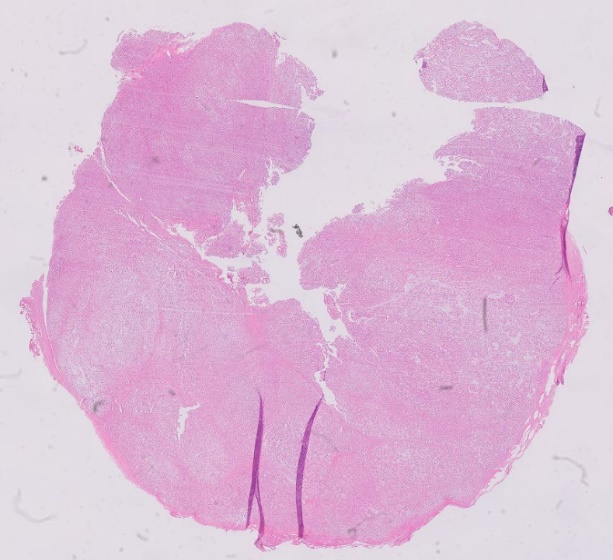
** **
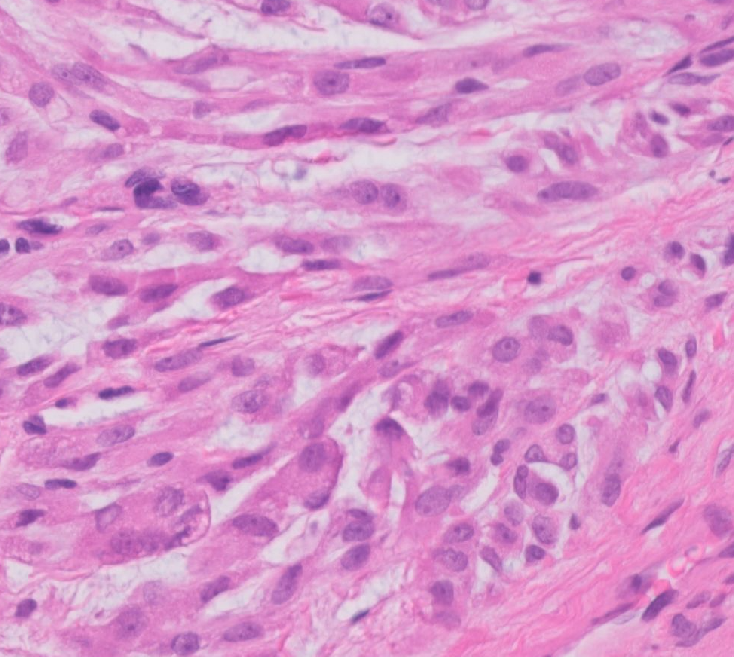
**

*Myoepithelial lesion with atypical features (case T11-01066)*

**
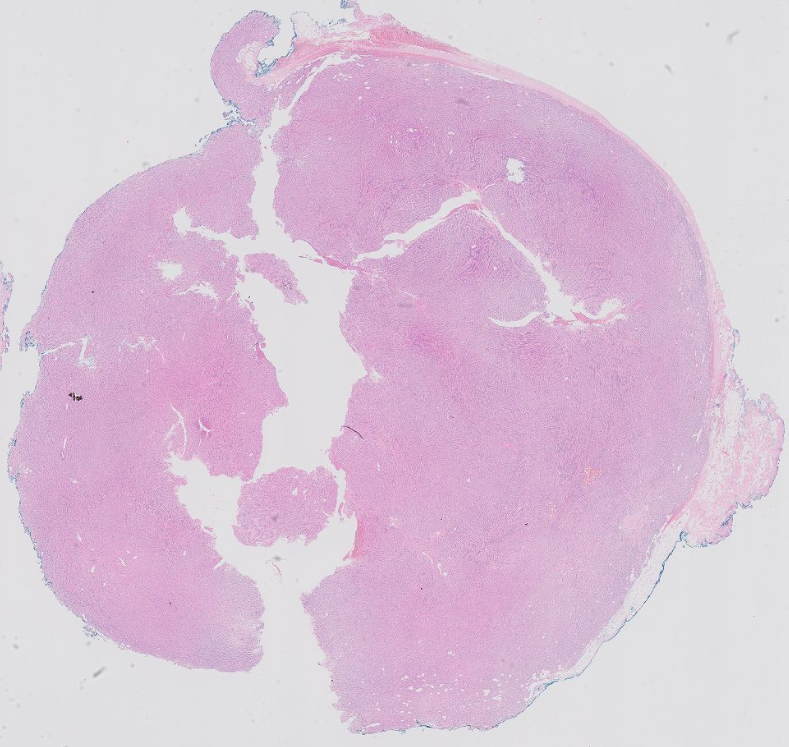
** **
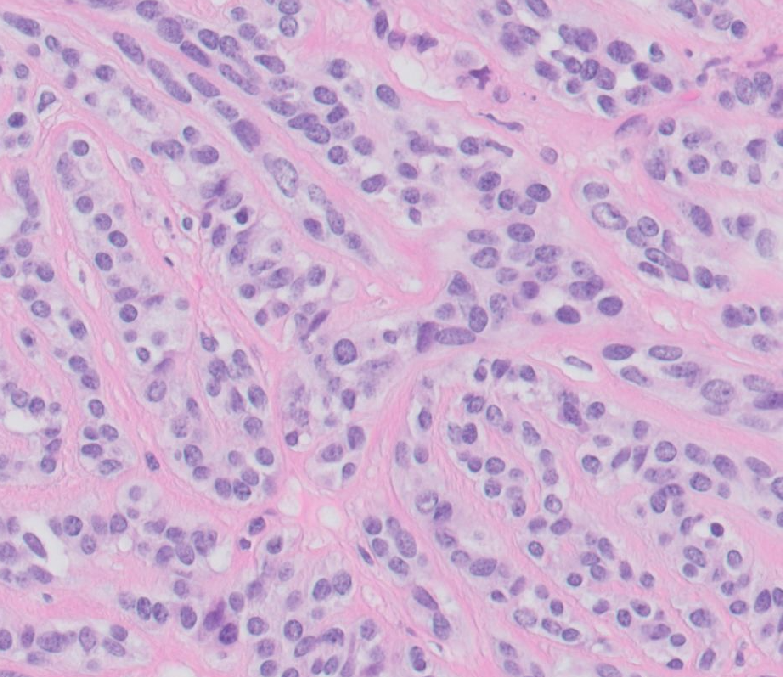
**

*Myoepithelial lesion with atypical features (case T11-01083)*

**
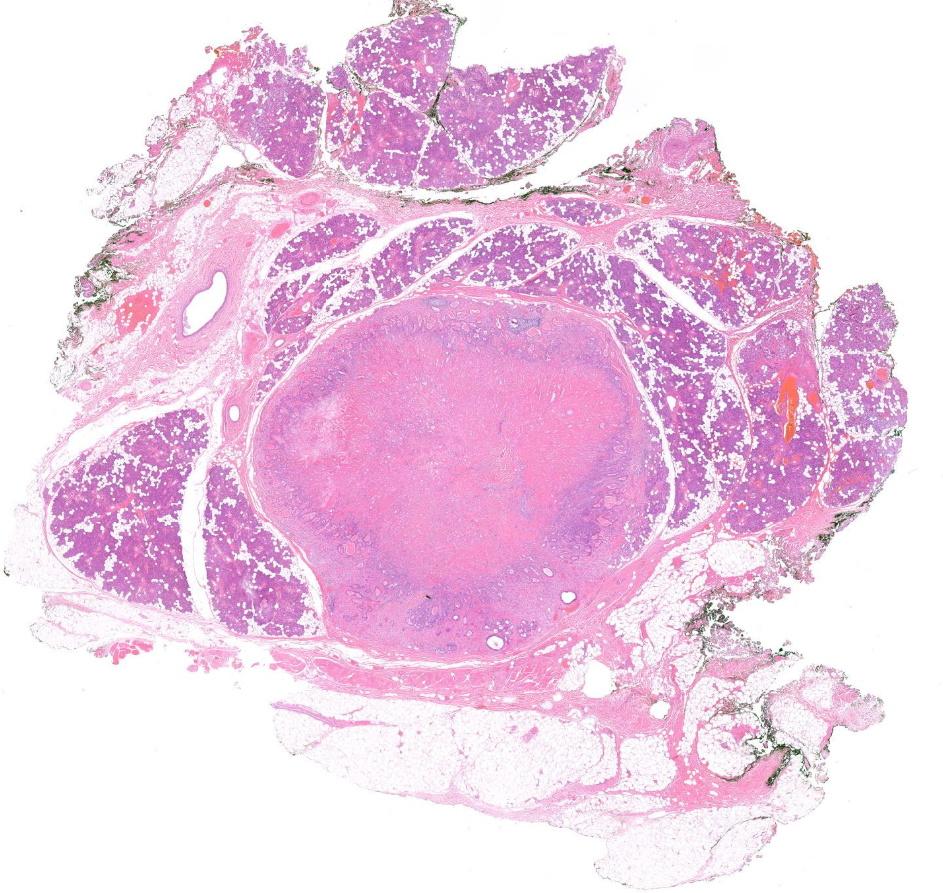
**
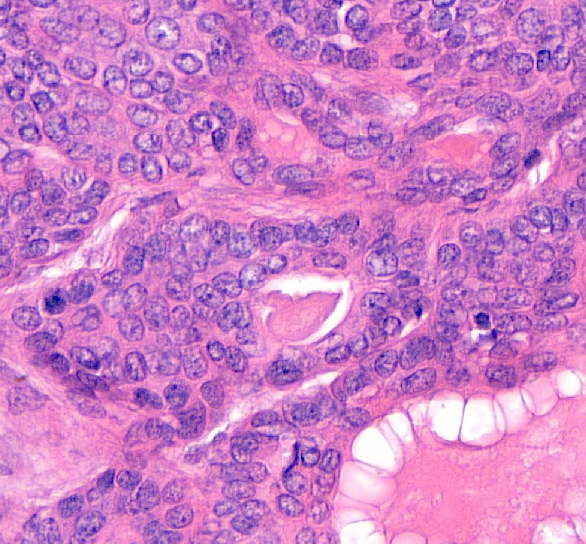
 **
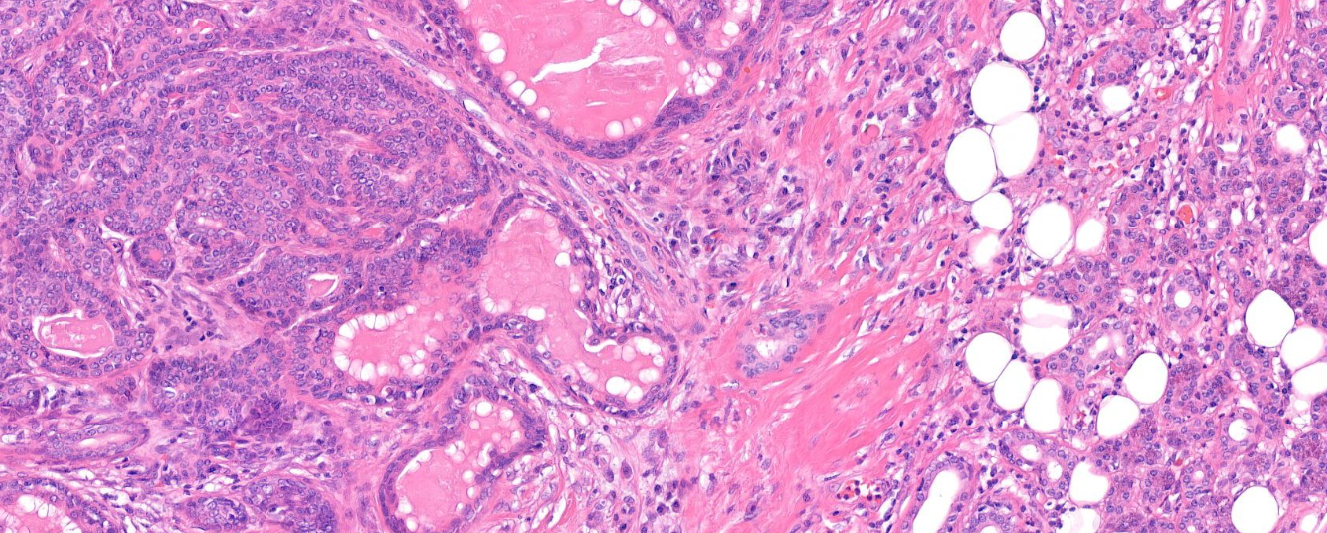
**
