# Supplementary material for: The SALV-Dataset Registry: An Expertly Curated Digital Clinicopathological Dataset for Salivary Gland Tumor Research and AI-Assisted Diagnostic Tools
Source: Head Neck Pathol. 2026 Jun 5;20(1):62. doi: 10.1007/s12105-026-01907-1 (PMC13241568; doi:10.1007/s12105-026-01907-1)
Supplement: Supplementary file 4 — (DOCX 555 kb) [file 12105_2026_1907_MOESM4_ESM.docx]

**Supplementary material 4**

Overview of the differential diagnoses suggested during team-based review, grouped by final consensus diagnosis. This figure shows the frequency and diversity of these suggested differentials per consensus entity, illustrating which tumor types were most frequently considered in the diagnostic process.

**
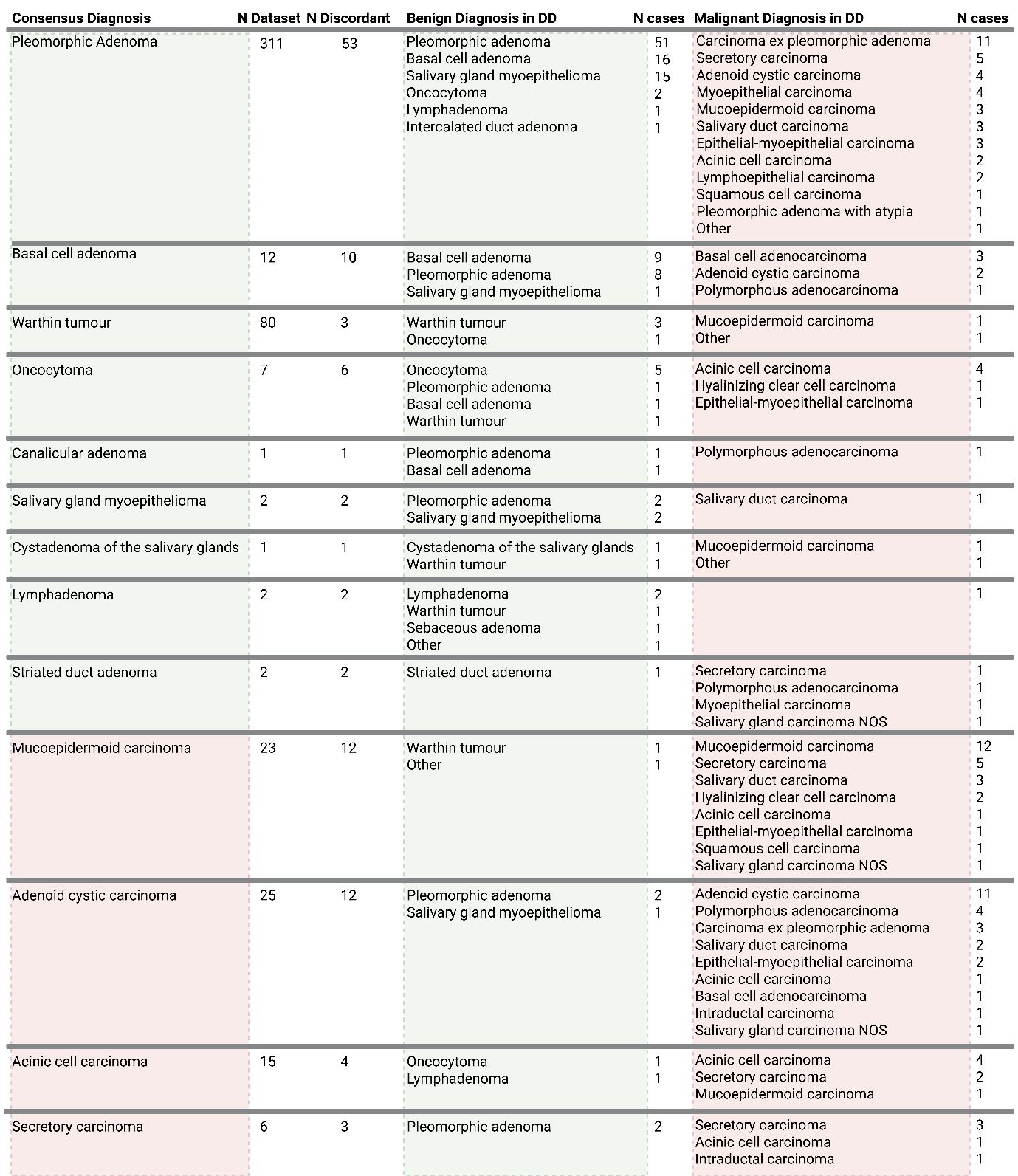
**

**
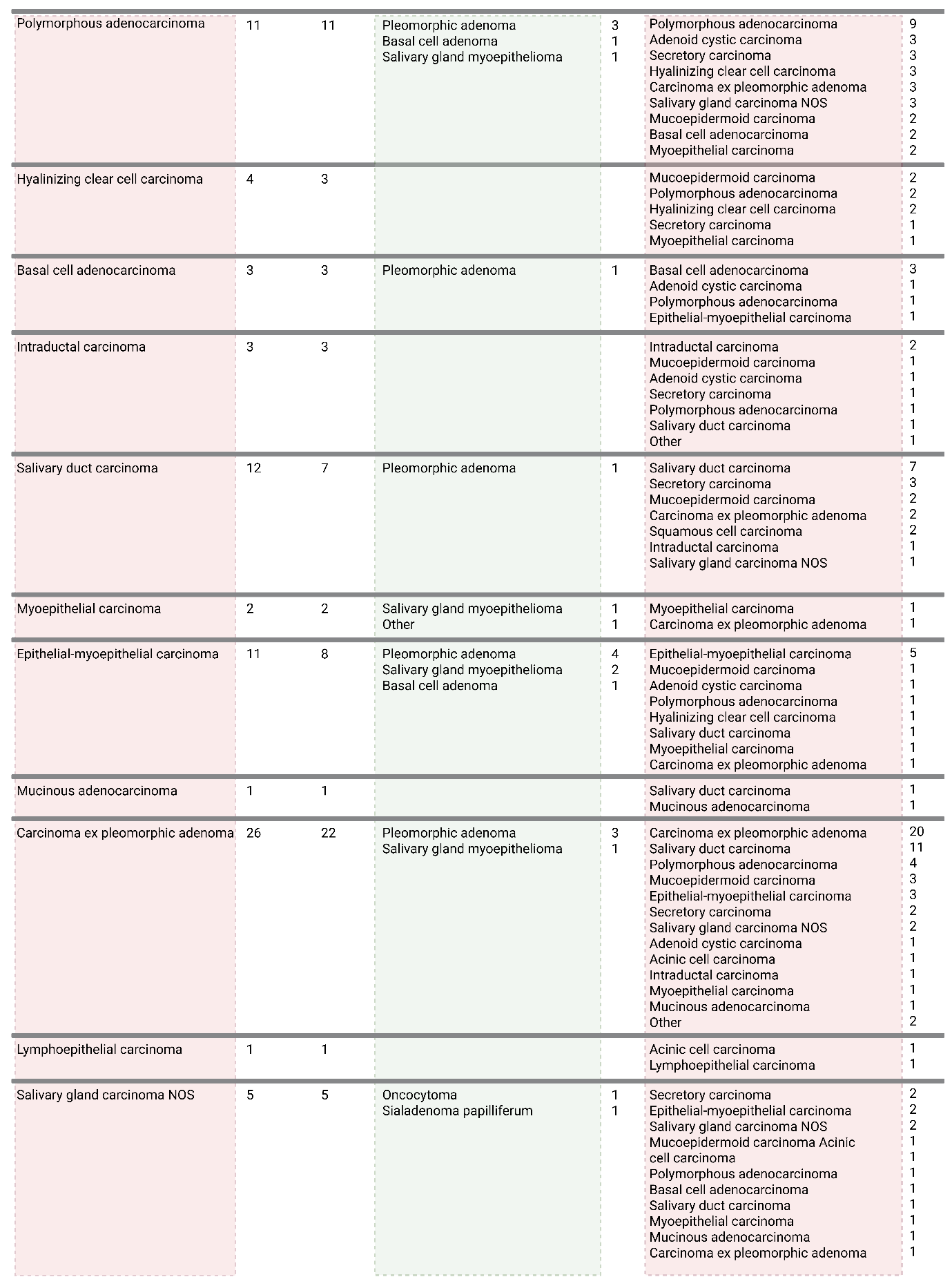
**
