# Supplementary material for: The SALV-Dataset Registry: An Expertly Curated Digital Clinicopathological Dataset for Salivary Gland Tumor Research and AI-Assisted Diagnostic Tools
Source: Head Neck Pathol. 2026 Jun 5;20(1):62. doi: 10.1007/s12105-026-01907-1 (PMC13241568; doi:10.1007/s12105-026-01907-1)
Supplement: Supplementary file 5 — (DOCX 24 kb) [file 12105_2026_1907_MOESM5_ESM.docx]

**Supplementary material 5.** Overview of discordant cases and rationale for diagnostic reclassification.

| **Original diagnosis** | **Revised diagnosis** | **Diagnostic change based on** |
| --- | --- | --- |
| *Benign tumors with change in subtype* |  |  |
| 1. Basal cell adenoma | Pleomorphic adenoma | Re-evaluation of morphology, β-catenin nuclear negative. |
| 2. Basal cell adenoma | Pleomorphic adenoma | Re-evaluation of morphology, β-catenin nuclear negative. |
| 3. Benign oncocytic tumour | Pleomorphic adenoma (atypical, oncocytic) | *LOC101928323*::*PLAG1* rearrangement. |
| 4. Pleomorphic adenoma | Basal cell adenoma | β-catenin nuclear positive. |
| 5. Basal cell adenoma | Pleomorphic adenoma (myoepithelial) | Re-evaluation of morphology. |
| 6. Myoepithelioma | Pleomorphic adenoma (myoepithelial) | Re-evaluation of morphology, biphasic staining pattern (p63, S100). |
| 7. Basal cell adenoma | Pleomorphic adenoma | Re-evaluation of morphology, no molecular alterations detected. |
| 8. Pleomorphic adenoma | Myoepithelioma | Re-evaluation of morphology, absence of biphasic staining pattern (p63, S100, calponin). |
| *Diagnostic change from benign to malignant* |  |  |
| 1. Cystadenoma | Cystadenocarcinoma (papillary) | Re-evaluation of morphology. |
| 2. Pleomorphic adenoma | Epithelial-myoepithelial carcinoma | Re-evaluation of morphology, *HRAS* and *PIK3CA* mutation. |
| 3. Oncocytoma | Acinic cell carcinoma (oncocytic) | Re-evaluation of morphology, *OXR1*::*NR4A3* rearrangement. |
| 4. Basal cell adenoma | Adenoid cystic carcinoma (sclerotic) | Re-evaluation of morphology, distinct biphasic tumor, no alterations detected. |
| 5. Basal cell adenoma | Epithelial-myoepithelial carcinoma (basaloid) | Re-evaluation of morphology, *HRAS* mutation. |
| *Diagnostic change from malignant to benign* |  |  |
| 1. Carcinoma ex pleomorphic adenoma | Pleomorphic adenoma (atypical, canalicular-like) | Re-evaluation of morphology, *HMGA2*::*WIF1* rearrangement and *MDM2* amplification. |
| 2. Carcinoma ex pleomorphic adenoma | Pleomorphic adenoma (atypical) | Re-evaluation of morphology. |
| 3. Adenocarcinoma NOS | Striated duct adenoma | Re-evaluation of morphology*, IDH2* mutation. |
| 4. Myoepithelial carcinoma | Pleomorphic adenoma (atypical) | Re-evaluation of morphology, *HMGA2*::intergenic rearrangement. |
| 5. Basal cell adenocarcinoma | Basal cell adenoma (membranous type with micro-invasion) | Re-evaluation of morphology and IHC, RNA quality insufficient for further analysis. |
| 6. Epithelial-myoepithelial carcinoma | Canalicular adenoma | Re-evaluation of morphology, absence of biphasic staining pattern, no alterations detected. |
| *Malignant tumors with change in subtype* |  |  |
| 1. Acinic cell carcinoma | Secretory carcinoma | *ETV6* rearrangement detected by FISH |
| 2. Mucoepidermoid carcinoma | Salivary duct carcinoma | Re-evaluation of morphology, androgen receptor IHC nuclear positive. |
| 3. Salivary gland carcinoma NOS | Hyalinizing clear cell carcinoma | *EWSR1*::*ATF1* rearrangement. |
| 4. Mucoepidermoid carcinoma (high grade) | Carcinoma ex pleomorphic adenoma (carcinoma NOS) | Re-evaluation of morphology and IHC. |
| 5. Basal cell adenocarcinoma | Carcinoma ex pleomorphic adenoma (carcinoma NOS) | Re-evaluation of morphology, RNA overexpression of *HMGA2* in sequencing analysis, no rearrangement detected. |
| 6. Carcinoma ex pleomorphic adenoma | Salivary gland carcinoma NOS | Re-evaluation of morphology and IHC. |
| 7. Adenoid cystic carcinoma | Polymorphous adenocarcinoma | Re-evaluation of morphology, p63 positive/p40 negative, *PRKD1* hotspot mutation detected. |
| 8. Salivary gland carcinoma NOS | Mucinous adenocarcinoma | Re-evaluation of morphology, NKX3.1 IHC positive. |
| 9. Adenoid cystic carcinoma, carcinoma NOS | Basal cell adenocarcinoma | Re-evaluation of morphology and IHC. |
| 10. Adenoid cystic carcinoma | *Multiple recurrences of the same patient. (carcinoma NOS) | Re-evaluation of morphology and IHC, no alterations detected. |
| 11. Carcinoma ex pleomorphic adenoma, intraductal carcinoma | Carcinoma ex pleomorphic adenoma (carcinoma NOS) | Re-evaluation of morphology, infiltrative growth. |
| 12. Salivary duct carcinoma | *Multiple recurrences of the same patient. | Re-evaluation of morphology. |
| 13. Carcinoma ex pleomorphic adenoma, adenoid cystic carcinoma | Adenoid cystic carcinoma | Re-evaluation of morphology, no alterations detected. |
| 14. Secretory carcinoma | Carcinoma ex pleomorphic adenoma (secretory carcinoma), DD collision tumor pleomorphic adenoma/secretory carcinoma | Partly biphasic IHC at lesion border (p63, SOX10), *ETV6*::*NTRK3* rearrangement. |
| *Change to or from an uncertain biological behavior* |  |  |
| 1. Pleomorphic adenoma* | Pleomorphic adenoma (atypical, myoepithelial, recurring) | Primary tumor (2012), re-evaluation of morphology. |
| 2. Pleomorphic adenoma* | Pleomorphic adenoma (atypical, myoepithelial, recurring) | First recurrence (2017), re-evaluation of morphology, ki-67 up to 20%. |
| 3. Pleomorphic adenoma* | Pleomorphic adenoma (atypical, myoepithelial, recurring) | Second recurrence (2020), re-evaluation of morphology. |
| 4. Pleomorphic adenoma* | Pleomorphic adenoma (atypical, myoepithelial, recurring) | Third recurrence (2022), re-evaluation of morphology, *NCALD*::*PLAG1* rearrangement. |
| 5. Myoepithelioma | Atypical myoepithelial lesion | Re-evaluation of morphology, *FGFR1*::*PLAG1* rearrangement. |
| 6. Pleomorphic adenoma, carcinoma ex pleomorphic adenoma | Atypical myoepithelial lesion | Re-evaluation of morphology, *NCALD*::*PLAG1* rearrangement. |
| 7. Myoepithelial carcinoma | Atypical myoepithelial lesion | Re-evaluation of morphology, *HMGA2*::chr12:66700226 rearrangement, *CDK4* amplification. |
| 8. Epithelial-myoepithelial carcinoma | Atypical myoepithelial lesion | Re-evaluation of morphology, *HMGA2*::*LOC105375977* rearrangement. |
| 9. Sialadenoma papilliferum, mucoepidermoid carcinoma | Salivary gland carcinoma NOS | Re-evaluation of morphology, no molecular alterations detected. |
| 10. Myoepithelial tumor | Pleomorphic adenoma, myoepithelial | Re-evaluation of morphology. |

**Multiple recurrences of the same patient.*
